# Primary cerebral cystic echinococcosis in a child from Roman countryside: Source attribution and scoping review of cases from the literature

**DOI:** 10.1371/journal.pntd.0011612

**Published:** 2023-09-05

**Authors:** Adriano Casulli, Stefania Pane, Franco Randi, Paola Scaramozzino, Andrea Carvelli, Carlo Efisio Marras, Andrea Carai, Azzurra Santoro, Federica Santolamazza, Francesca Tamarozzi, Lorenza Putignani

**Affiliations:** 1 WHO Collaborating Centre for the Epidemiology, Detection and Control of Cystic and Alveolar Echinococcosis, Department of Infectious Diseases, Istituto Superiore di Sanità, Rome, Italy; 2 European Union Reference Laboratory for Parasites, Department of Infectious Diseases, Istituto Superiore di Sanità, Rome, Italy; 3 Unit of Microbiomics, Bambino Gesù Children’s Hospital, IRCCS, Rome, Italy; 4 Neurosurgery Unit, IRCCS Bambino Gesù Children’s Hospital, Rome, Italy; 5 Istituto Zooprofilattico Sperimentale del Lazio e della Toscana “*M*. *Aleandri*”, Epidemiology Unit, Rome, Italy; 6 Department of Infectious-Tropical Diseases and Microbiology, WHO Collaborating centre on Strongyloidiasis and other Neglected Tropical Diseases, IRCCS Sacro Cuore Don Calabria Hospital, Negrar di Valpolicella, Verona, Italy; 7 Unit of Microbiomics; Research Unit of Human Microbiome, Bambino Gesù Children’s Hospital, IRCCS, Rome, Italy; FRANCE

## Abstract

**Background:**

Human cystic echinococcosis (CE) is a zoonotic parasitic infection caused by the larval stage of the species belonging to the *Echinococcus granulosus sensu lato* (*s*.*l*.) complex. Parasitic cysts causing human CE are mainly localized in the liver and in the lungs. In a smaller number of cases, larvae may establish in any organ or tissue, including the central nervous system (CNS). Cerebral CE (CCE) is rare but poses serious clinical challenges.

**Methods:**

This study presents a case of CCE in a child living in the countryside near Rome (Italy), along with a comparative molecular analysis of the isolated cyst specimens from the patient and sheep of local farms. We also systematically searched the literature to summarize the most relevant epidemiological and clinical aspects of this uncommon localization.

**Findings:**

The comparative molecular analysis confirmed that the infection was caused by *E*. *granulosus sensu stricto* (*s*.*s*.) (G3 genotype), and most likely acquired in the family farm.

The literature search identified 2,238 cases of CCE. In 80.51% of cases, brain was the only localization and single CCE cysts were present in 84.07% of cases. Mean patients’ age was 20 years and 70.46% were children. Cyst rupture was reported in 12.96% and recurrence of CCE after treatment in 9.61% of cases. Permanent disability was reported in 7.86% of cases, while death occurred in 6.21%. In case series reporting all CE localization, CCE represented 1.5% of all CE cases. In the few reports that identified at molecular level the CCE cyst, *E*. *granulosus s*.*s*. was found in 40% and *E*. *canadensis* in 60% of cases.

**Conclusions:**

We report a rare case of CCE and evidenced the probable local origin of infection. The proportions of CE cases with uncommon localizations and with high impact on patients’ lives have been globally neglected and should be included in the computation of the global burden of CE.

## Author summary

Human cystic echinococcosis (CE) is a parasitic zoonotic disease caused by cestode species belonging to *Echinococcus granulosus s*.*l*. complex. CE affects mainly pastoral and rural communities in both low-income and upper-middle-income countries, where the life cycle of the parasite is sustained between livestock and dogs. The larval parasite cysts causing human CE develop mainly in the liver and secondarily the lung, but in a smaller number of cases, larvae may develop in any organ or tissue, including the brain.

We report here a case of cerebral CE (CCE) in a child living near Rome (Italy), which was attributed to probable household/backyard infection from the family farm by comparative molecular analysis of the patient’s and sheep CE cyst specimens obtained from the family farm and another local farm. We also summarized the clinical characteristics of CCE cases retrieved through a literature review. When considering clinical centres reporting all anatomical sites of CE, liver represented 70%, lungs 19%, and unusual localizations 11% of all CE cases. The proportion of CCE among all CE cyst localizations represented 1.5% of all CE cases. These figures are important when calculating the global burden of disease, since CCE and other uncommon CE localizations, although more rare, are clinically more severe.

## Introduction

Echinococcosis is one of the current 20 disease groups prioritized for control by the World Health Organization (WHO) within the umbrella of the Neglected Tropical Diseases (NTDs) [1, 2]. Within this disease group, cystic echinococcosis (CE) caused by *Echinococcus granulosus sensu lato* (*s*.*l*.) and alveolar echinococcosis (AE) caused by *Echinococcus multilocularis*, are those of major human interest for their impact on patients’ and global public health. *E*. *granulosus s*.*l*. is a complex of species and genotypes encompassing *Echinococcus granulosus sensu stricto* (*s*.*s*.) (genotypes G1 and G3, also known as the “sheep and buffalo strains”), *Echinococcus equinus* (genotype G4, “horse strain”), *Echinococcus ortleppi* (genotype G5, “cattle strain”) and *Echinococcus canadensis* cluster (genotypes G6/7, “camel and pig strains”; genotypes G8/G10, “cervid strains”) [3, 4]. Among these two human diseases, CE is the most prevalent and worldwide distributed due to the synantropic life cycle of *E*. *granulosus s*.*l*. in rural areas, that mainly involves livestock as intermediate hosts, most commonly sheep, but also cattle and pigs, and dogs as definitive hosts [3].

Humans, dead-end accidental hosts, can be infected by the ingestion of *E*. *granulosus s*.*l*. eggs excreted by infected dogs with faeces that contaminate the environment. From the infection event to the onset of symptoms, that are not specific, it may pass an indefinite period of time, from a few months to years (or even lifelong in completely asymptomatic infections). This long incubation period makes virtually impossible to track back the event of infection for a better understanding of the different pathways of transmission and identification of the real risk factors for CE. Human CE has been historically considered as a foodborne disease and a disease associated with “contact with dogs”. Currently, three main drivers of infection have been highlighted: hand-to-mouth, foodborne and waterborne transmissions [5]. However, it is not clear what is the relative contribution of these three transmission routes for human infections. A recent systematic review on source attribution of human CE and AE, pointed out that hand-to-mouth after dog contact and waterborne routes may play a major role in the transmission of CE [6].

CE is a chronic disease with a low fatality rate, characterised by anatomically isolated, fluid-filled parasitic cysts growing concentrically in organs and tissues. CE cysts cause symptoms, when this occurs, due to mass-effect on surrounding organs and structures. Complications may occur due to cyst rupture (biliary obstruction, bronchial obstruction, vascular embolism, haemorrhage, anaphylactic reactions and secondary echinococcosis due to dissemination of protoscoleces contained in the spilled fluid) and bacterial super-infection of the cysts [7]. Patients suffering from CE usually have single organ involvement, with the presence of a single cyst and less commonly multiple cysts involving single or multiple organs [7]. CE cysts are mainly localized in the liver and secondarily in the lungs. In a smaller number of cases, considered unusual localizations for CE (UL-CE), larvae may establish in any organ or tissue, including the central nervous system (CNS). CNS-CE includes cerebral (CCE) and spinal localizations. CCE cases pose serious clinical concern [8, 9]. Treatment of CCE may be complicated by intraoperative rupture of cysts, often leading to long-term sequelae, permanent disability or eventually death [8, 9].

In this study, we present a rare case of CCE in a child living in the countryside at the outskirts of Rome (Italy) along with a comparative molecular analysis of the patient’s and sheep CE cyst specimens obtained from the family and another local farm. We also systematically reviewed published case reports and case series of CCE to summarize the most relevant epidemiological and clinical aspects of this uncommon localization.

### Case description

**Fig 1:**
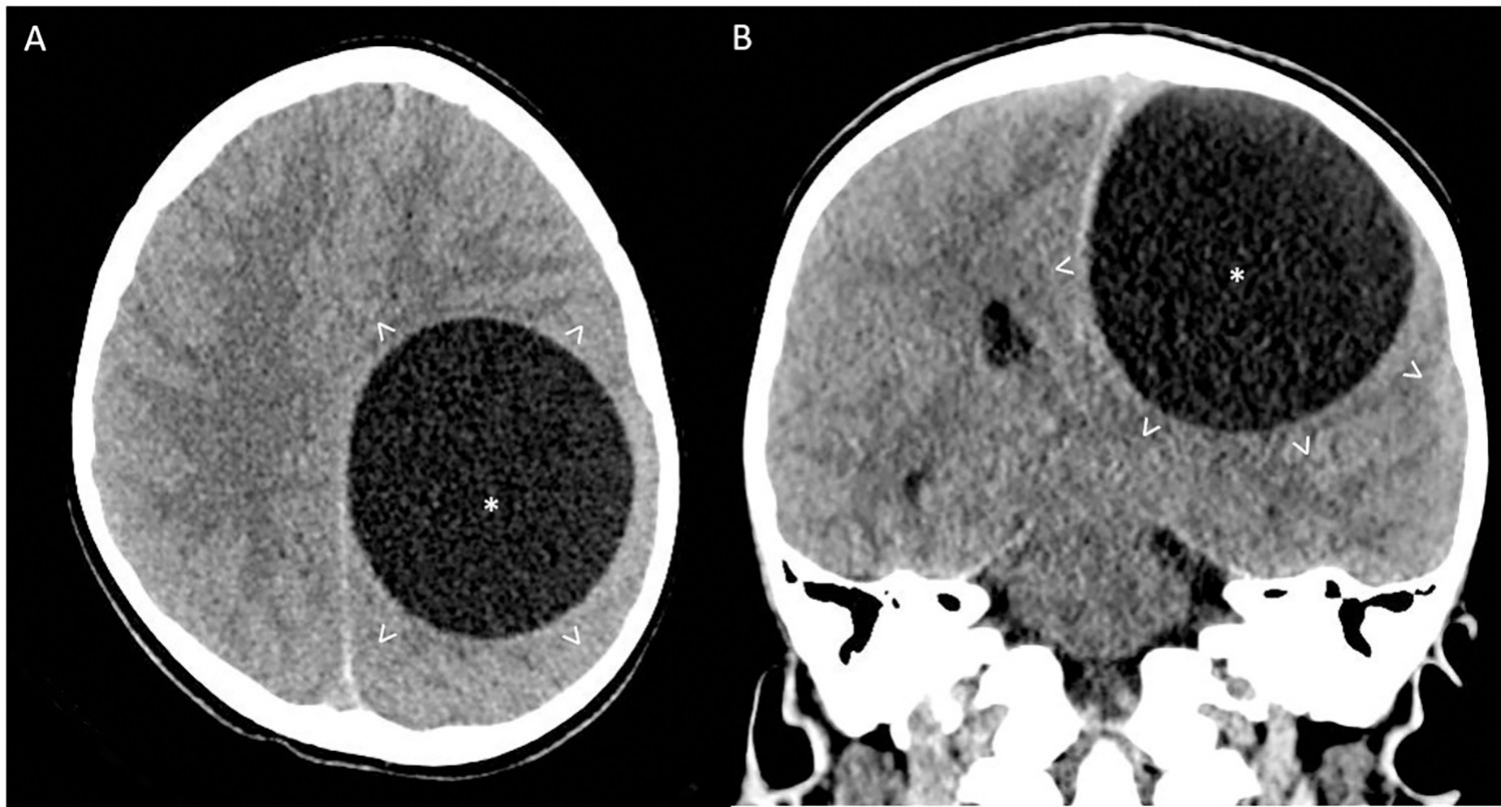
Computed tomography imaging without contrast obtained at presentation. Axial (A) and coronal (B) planes are shown. There is evidence of a round-shaped hypodense left frontal-parietal lesion (*) determining significant mass effect and no evidence of perilesional edema (arrowheads).

**Fig 2:**
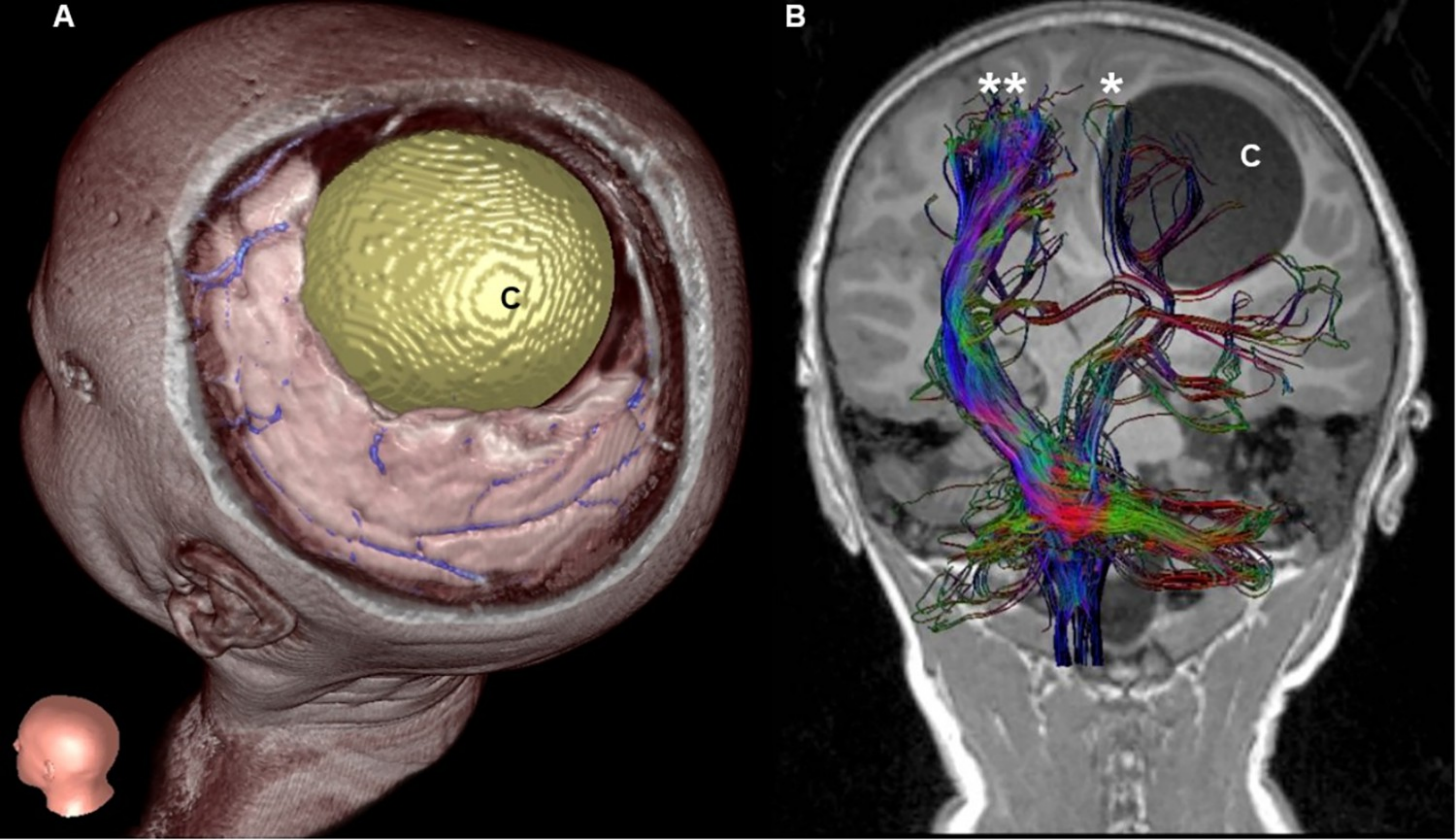
A) Tridimensional reconstruction of the left frontal-parietal cystic lesion determining massive brain compression. B) T1 weighted Magnetic Resonance imaging, coronal section integrated by diffusion tensor imaging and 3D fiber tract reconstruction. The cystic lesion (C) compression on the left cortical-spinal tract (*) is shown in comparison to the controlateral cortical-spinal tract (**).

In April 2019, a 6-year-old boy living in the family farm in a rural area near Marino (Rome province, Lazio region, Italy) was brought to the Bambino Gesù Children’s Hospital in Rome with a history of recurrent, worsening headache episodes since February 2019 and recent onset of right hemiparesis, occurred two weeks before hospitalization. Computed tomography (CT) (Fig 1) revealed an intracerebral hypodense lesion and the patient was subsequently admitted to the neurosurgical ward for in-depth investigations and treatment. An examination of the fundus oculi showed findings compatible with intracranial hypertension. Magnetic resonance imaging (MRI) (Fig 2) confirmed a left frontal-parietal expansive cystic lesion measuring 7x7.5x6 cm in size, with a cerebrospinal fluid-like content, no peripheral edema, and no contrast enhancement, determining mass-effect on surrounding brain structures.

The differential diagnosis included neuroepithelial and parasitic cyst. Abdominal ultrasound and chest x-ray were unremarkable. Laboratory results only showed a slightly elevated C-reactive protein (CRP) (6.27 mg/L; n.v. < 0.5 mg/dL) and a mild neutrophilia (13.2 10^3^/μL; n.v. 1.7–8.3 10^3^/μL) (most likely an concomitant finding unrelated to the reason for attention); there was no eosinophilia.

Serology was negative for *Taenia solium* (performed out of precaution although the characteristics of the lesion were little evocative; Taenia solium IgG NovaLisa ELISA kit, NovaTec Immundiagnostica GmbH, Dietzenbach, Germany), *Strongyloides* spp. (performed in anticipation of possible need of steroidal therapy; after treatment Strongyloïdes ratti IgG ELISA kit, Bordier Affinity Products Crissier, Switzerland) and *Echinococcus* spp. by indirect hemagglutination (IHA) (Cellognost Echinococcosis, Siemens, Erlangen, Germany).

**Fig 3:**
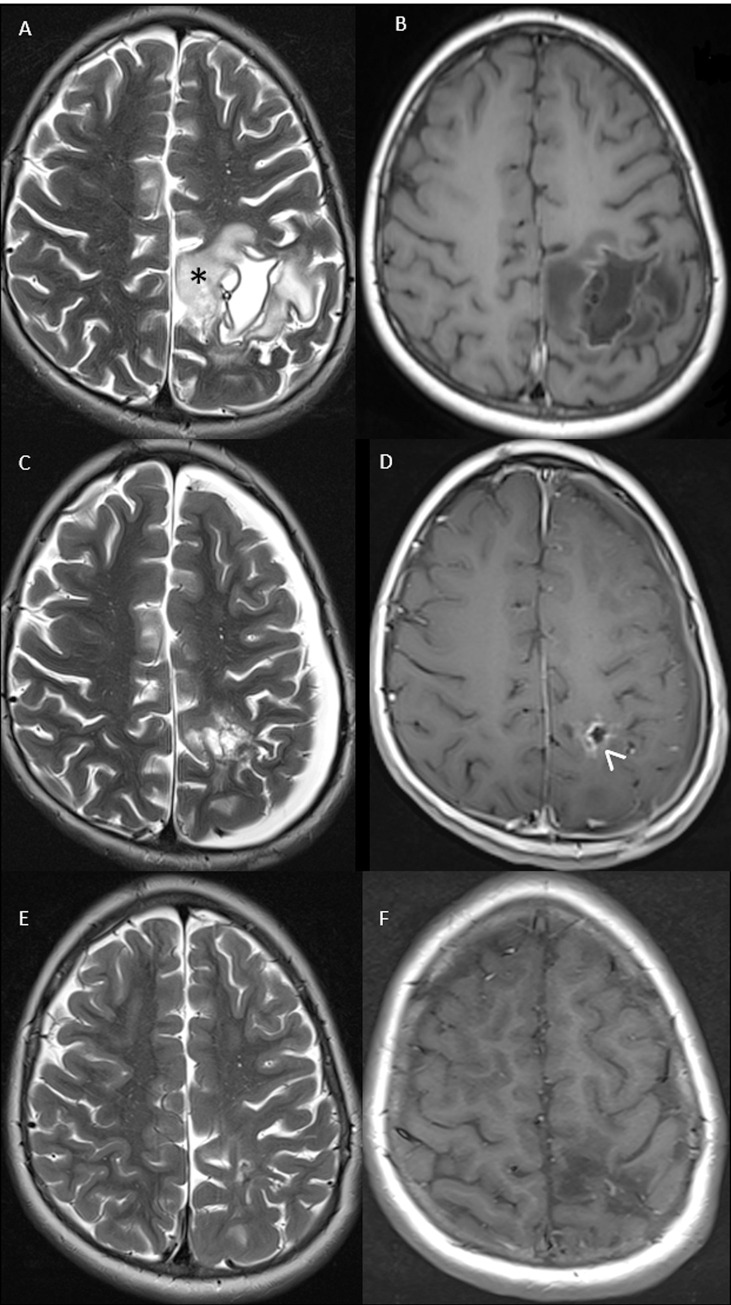
T1 (A, C, E) and T2 (B, D, F) weighted Magnetic Resonance imaging one month after stereotactic cyst drainage (A, B) demonstrated collapse of the cystic cavity (+) and perilesional edema (asterix). Follow-up imaging after 4 weeks of corticosteroid treatment (C, D) demonstrated regression of edema and linear contrast enhancement of the surgical cavity (arrowhead). Long term follow-up (E, F), 12 months after surgery, showed complete disappearance of the lesion.

**Fig 4:**
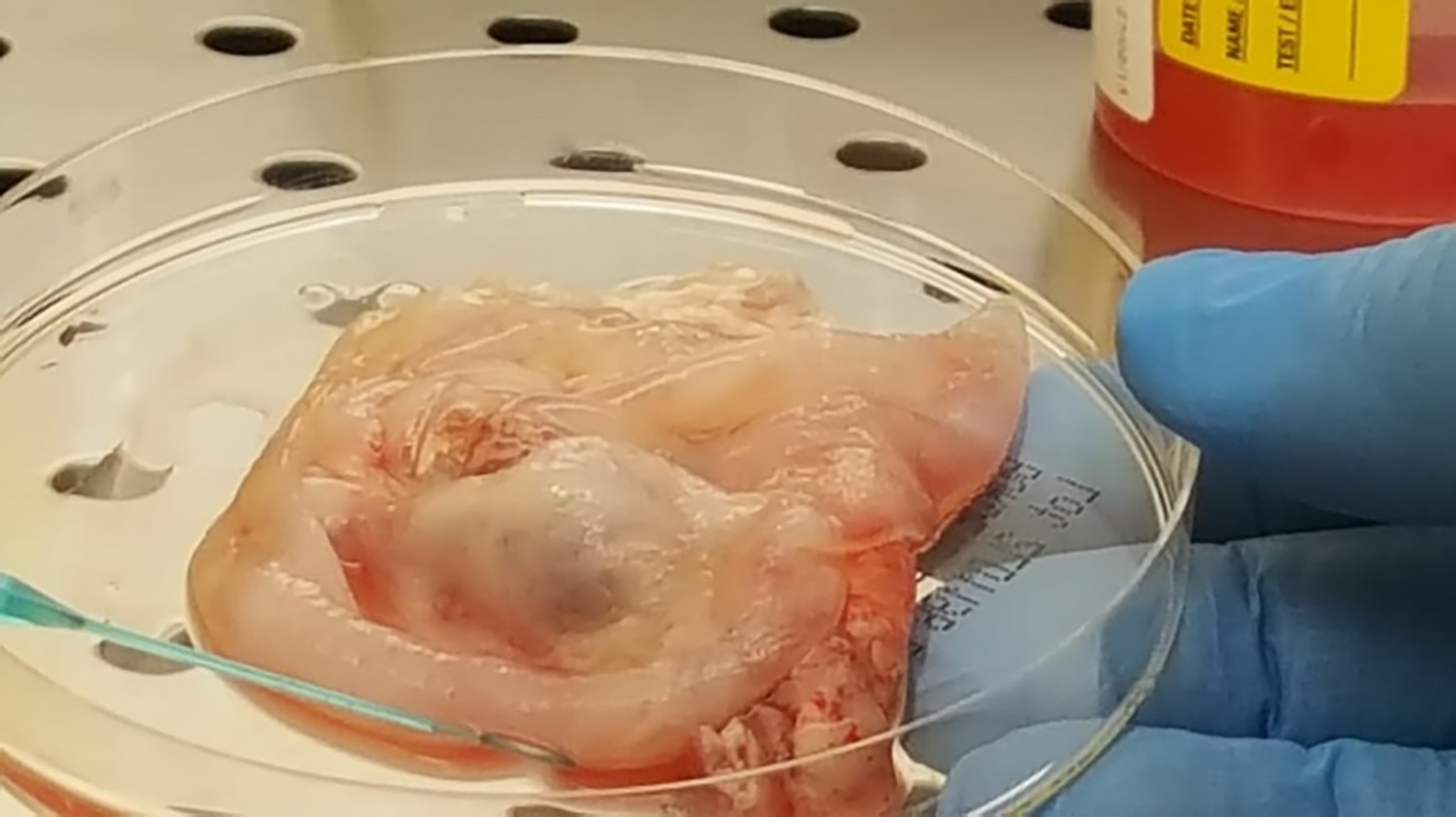
Cerebral echinococcal cyst removed from the patient and used for molecular diagnosis and *Echinococcus granulosus sensu lato* species identification.

Treatment with albendazole (15 mg/kg in two divided doses) was started as a precaution before invasive procedures and etiological diagnosis of the lesion, due to frequent negativity of serology in extra-hepatic CE. In order to drain intracystic fluid and to reduce brain compression, an intracystic catheter was positioned with the aid of intraoperative image-guidance; the catheter was then connected to a subcutaneous reservoir (Rickham reservoir) to allow subsequent drainage of cystic fluid. This was macroscopically corpusculated and negative at microscopy and for the presence of neoplastic cells. After transient clinical benefit, worsening of the right motor weakness in combination with self-resolving focal motor epileptic seizures lasting about two minutes and worsening of headache were observed. Hence, antiepileptic therapy with carbamazepine was started. CT and MRI showed resolution of mass effect and development of perilesional edema. Particularly, MRI one month after stereotactic cyst drainage (Fig 3, panels A, B) showed collapse of the cystic cavity and presence of perilesional edema. Hence, corticosteroid treatment by dexametasone was started for 4 weeks. After 3 weeks of treatment, MRI examination showed persistence of perilesional cerebral edema. The child underwent a left frontal-parietal craniotomy and microsurgical removal of the lesion (Fig 4).

**Fig 5:**
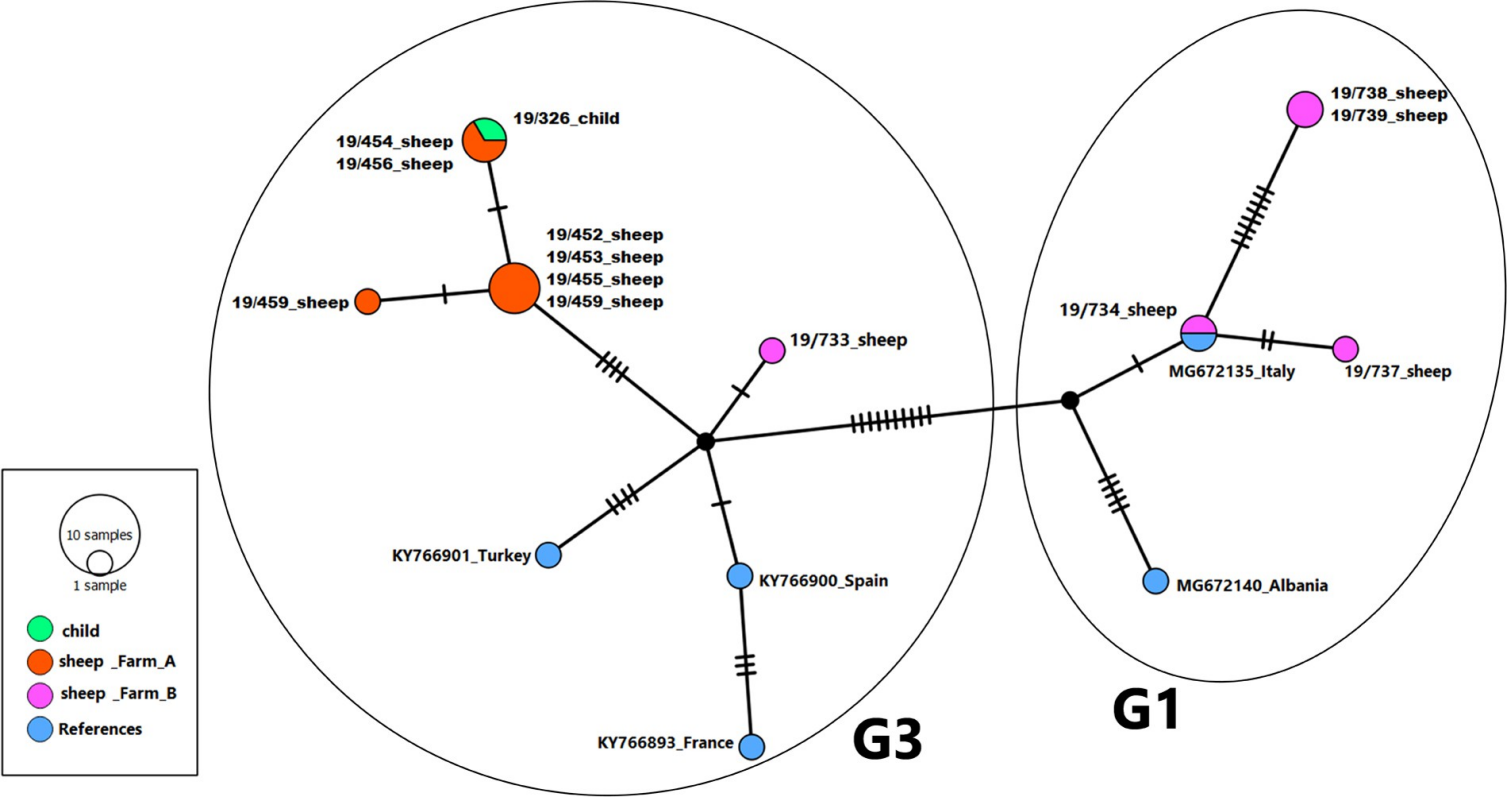
Haplotype network of the concatenated sequence fragments (total 2,368 bp) of the genes COX1, NAD5, NAD2 and NAD1 from 13 samples of *Echinococcus granulosus* including cyst removed from the child (green), sheep cysts from farm A (orange) and sheep cysts from farm B (pink), and five reference sequences retrieved from *E*. *granulosus* reference mitochondrial genomes (blue) from France, Spain, Turkey (G3), Albania and Italy (G1) (GenBank Accession numbers: KY766900, KY766901, KY766903, MG672135, MG672140).

The fluid content of the cyst was centrifuged and stained with hematoxylin/eosin. Microscopic examination showed *E*. *granulosus* parasitic elements (so called “hydatid sand”), allowing a conclusive diagnosis of CE. An aliquot of the cyst wall was analyzed at the European Union Reference Laboratory for Parasites (EURLP, Istituto Superiore di Sanità, Rome, Italy) by PCR and sequencing of a fragment of the COX1 mitochondrial gene [10], which allowed identification at genotype level of *Echinococcus granulosus s*.*l*. (Fig 5). Therapy with albendazole was continued until July 2019 for a total of 3 months. Despite the possible effect of carbamazepine of albendazole metabolism, dosage of plasma levels of albendazole sulphoxide was not carried out due to the unavailability of this service. The follow-up imaging demonstrated regression of the edema and linear contrast enhancement of the surgical cavity (Fig 3, panels C, D). Follow-up with imaging, 12 months after surgery, showed complete disappearance of the lesion (Fig 3, panels E, F). Follow-up with serology is not recommended in CE; serology was performed after surgery out of completeness since seroconversion may occur after loss of integrity of the cyst. However, it persisted negative. Anti-epileptic therapy with carbamazepine was discontinued in October 2020 and EEG was normal (last in March 2021) after initial and only temporary occurrence of left hemispheric slow EEG abnormalities early after intervention. His conditions were stable at the 30 month follow-up visit in December 2022, with absence of headache and seizures.

### Epidemiological investigation and source attribution by molecular approach

In Italy, echinococcosis is a notifiable disease both in humans and animals. No active human surveillance is implemented, and cases notification de facto only relies on Hospital Discharge Records. In livestock, CE surveillance is passive, mainly based on compulsory *post-mortem* slaughterhouse inspection of susceptible species. After an animal CE outbreak is confirmed and notified, control measures including targeted antiparasitic treatment of dogs and biosecurity measures are applied to the positive farm. In Lazio Region, where the case occurred, the median prevalence of CE in sheep was estimated to be 1.9% (95% CI: 0.8–4.1) [11]. From July to December 2019, seven echinococcal cyst samples, obtained from slaughtered sheep from the same farm owned by the child’s family (farm A), as well as five randomly collected cysts from slaughtered sheep raised in a different farm (farm B) located in the neighbourhood of the farm A and sharing the same pastures, were collected from the local abattoir and sent to EURLP for molecular analysis. Genomic DNA was extracted from small flaps of the cysts’ germinal layers with the DNeasy *Blood* & *Tissue* Kit (Qiagen) according to manufacturer’s instructions. All 13 cyst samples (from the child and from sheep of farms A and B) were identified as *Echinococcus granulosus s*.*s*. with an RFLP method [12] and DNA was used as template for the amplification of the partial mitochondrial genes COX1, NAD5, NAD2 and NAD1 [10, 13, 14]. The analysis of variable positions allowed identifying the cyst of the child and the cysts of the sheep from farm A as G3 genotype, while 4/5 and 1/5 of cysts of sheep from farm B as G1 and G3 genotypes, respectively. Homologous sequence fragments were retrieved from complete G1 and G3 mitochondrial genomes available in GenBank (KY766900, KY766901, KY766893, MG672135, MG672140) to assemble comparable reference sequences to be included in genetic analyses (Fig 5). Concatenated sequences (total 2,368 bp) were aligned and analysed to generate a TCS network using the software PopART (http://popart.otago.ac.nz). Results showed a clear and direct genetic relationship between the child’s cyst and the cysts belonging to sheep of his own family’s farm (farm A). In particular two sheep cyst sequences were identical (100%) to those of the child. Epidemiological investigation confirmed the past close contact and playing habits of the child with dogs living in the farm as well as playing habits on the ground in the courtyard and the presence of a kitchen garden from products of which the child was occasionally fed. The parents of the child were also tested by ultrasound and resulted negative to CE.

Several studies identified the species or genotypes of *E*. *granulosus s*.*l*. infecting humans but as far as we know, only one study explored the source of human infection by molecular approach to identify animal species causing the infection [15]. To our knowledge, this is the first time source attribution of human infection at local scale was attempted, by comparative molecular approach of concatenated sequences of *E*. *granulosus s*.*l*.

## Methods

### Literature search strategy and selection criteria

A scoping review was undertaken to identify clinical reports of CCE cases published in the literature. This search was aiming at the identification of epidemiological and clinical characteristics of CCE from case reports and case series. This review is reported according to PRISMA guidelines (S1 Fig). MEDLINE (PubMed), EMBASE (Excerpta Medica Database), SCI SEARCH (Science Citation Index), and GOOGLE SCHOLAR databases were screened for literature search up to December 30, 2022. No restriction to year of publication was applied. Only papers in English were included. Databases were searched using keywords associated with the Boolean operators AND and OR. The full electronic search strategy was: (cerebral OR cerebellar OR ventric* OR intraventricular OR Central Nervous System OR CNS OR unusual loc* OR uncommon loc* OR rare loc* OR pineal region OR intracranial OR cranial OR *brainstem* OR *brain stem* OR brain OR extrahepatic OR extra-hepatopulmonary) AND (cystic echinococcosis OR hydatid disease* OR Hydatid* OR echinococcal OR echinococcal cyst* OR Echinococcus granulosus OR Echinococcus canadensis) NOT (alveolar OR multilocularis OR E. multilocularis OR Echinococcus multilocularis).

The inclusion criterion was description of clinical records of confirmed human CCE diagnosed by pathognomonic features on imaging or laboratory confirmed by histology, microscopy, or molecular tests. Publications were excluded if they lacked original data (reviews without primary data) or included duplicated data between papers, or if did not concern CE, or concerned animal hosts. After duplicates between databases were removed, at least two independent researchers screened the title and abstract for potential eligibility. Disagreement was resolved by consensus between the researchers. The full texts of the potentially eligible papers were then examined to assess their eligibility and their references list assessed for additional potentially eligible papers. For each eligible paper, the following data were extracted using an Excel sheet: publication details, country of origin, year of presentation (when not available, year of publication), type and number of CE cases described in case cohorts (CCE, CNS-CE, UL-CE, any localization), demographic and clinical characteristics at presentation (sex, age or age group [≤ or > 18 years] where precise age was not available, CCE as sole localization or additional localizations also present, single or multiple CCE) and clinical course and outcome during clinical management (cyst rupture events, sequelae leading to permanent disability and deaths).

### Results from case-reports and case-series

The search identified 3,359 articles, of which 1,585 were excluded because duplicates. The remaining 1,776 were assessed for eligibility and 1,352 were excluded (S2 Fig). Thirteen additional articles were identified while screening the bibliography of eligible publications (S2 Fig). Data were eventually extracted from 437 publications describing a total of 2,238 cases of human CCE reported from 54 countries (S2 Fig and S1 Text). Most of the publications were from Turkey (N = 126; 28.83%), India (N = 75; 17.16%) and Iran (N = 45; 10.29%), but also from Morocco (N = 14), Spain (N = 13), China (N = 12), South Africa (N = 12), Italy (N = 11) and Tunisia (N = 11). Most papers were published from 2000 to 2022 (284/437; 64.99%), while the remaining were published from 1914 to 1999 (153/437; 35.01%), of which 69.93% (107/153) from 1980 to 1999 and 30.07% (46/153) from 1914 to 1979.

Of 1,448 cases for which data on age was present, 70.44% (n = 1,020) were children (≤18 years) and 29.56% (n = 428) were adults (>18 years). Mean age of children was 10 years (min 1; max 18), while mean age of adults was 34 years (min 19; max 82). When considering both age groups together, mean age of all CCE cases was 20 years. Of 1,690 cases for which data on sex was present, 56.51% (N = 955) were males and 43.49% (N = 735) were females.

Of 1,375 cases for which this data was available, 80.51% (N = 1,107) were primary cases of CCE (i.e. CCE was the sole cyst localization), while in 19.49% (N = 268) were secondary CCE cases where CE cysts were found also in other organs. Of 1,563 cases for which this data was available, single CCE cysts were detected in 84.07% (N = 1,314) of cases and multiple CCE cysts in 15.93% (N = 249). An episode of cyst rupture was reported in 12.96% (290/2,238) of the total documented CCE cases, while recurrence in 9.61% (215/2,238). Permanent disability was identified in 7.86% (176/2,238) of the total documented CCE cases. Death occurred in 6.21% (139/2,238) of CCE cases, in the majority of cases intra-operatory.

Considering only the eligible studies from clinical centres reporting any cases of CE in any anatomical localization and where it was possible to separate liver, lung, from all other localizations (N = 7), liver represented 69.99% (1,441/2,059) of single organ localizations and 71.59% (1562/2,182) when considering multiple localizations [16–22]. In the same case series, lungs represented 19.18% (395/2,059) of single organ localizations and 20.16% (440/2,182) when considering multiple localizations. In the same case series, all other UL-CE localization (different from liver and lungs) represented 10.83% (223/2,059) of all documented CE cases.

Considering only the eligible studies from clinical centres reporting any localization of CE where it was possible to separate CCE from all other anatomical sites (N = 48), CCE occurred in 1.90% (183/9,640) of all cases [16–63]. When considering only cohorts of adults (N = 32) from the previous case series reporting any localization of CE where it was possible to separate CCE from all other anatomical sites, CCE occurred in 1.50% (129/8,572) of all cases [16–26, 30, 34, 36–41, 43–46, 48, 50, 52–55, 58, 61, 62]. Considering only CNS-CE cohorts (N = 28), CCE represented 66.06% (N = 473/716) of these cases [22, 23, 30, 35, 37, 48, 62, 64–84]. Considering only cohorts of UL-CE localization (N = 18), CCE represented 14.16% (96/678) of cases [17, 19, 20, 22–26, 30, 37, 62, 85–91].

For what concerns species belonging to *E*. *granulosus s*.*l*. complex causing CCE, only 20 cases were molecularly confirmed in 7 studies and result identified as *E*. *granulosus s*.*s*. in 40% of cases (n = 4 were reported as genotype G1, n = 3 as G2 genotype and n = 1 as unspecified genotype) and *E*. *canadensis* in 60% of cases (n = 3 were reported as G6/7 genotype cluster and n = 9 as G6 genotype) [29, 61, 92–96].

No additional clinical data could be summarized since most papers (N = 437) lacked adequate information or clear description of clinical symptoms, diagnostic procedures, treatment options, outcome and follow-up of CCE cases.

## Discussion

### Clinical epidemiology of CCE

After *E*. *granulosus s*.*l*. eggs are ingested by humans, hatched embryos (oncospheres) penetrate the gut wall and enter in the circulation and then reach internal organs, where they may develop into the larval stage (metacestode) causing CE. In particular, through the portal system, embryos reach the liver first. Embryos passing this barrier, through the right side of the heart, reach then the lungs. Rarely embryos establish in different organs and tissues. The factors influencing the rate of growth of the cyst are largely unknown, but likely related to the space that the cyst can occupy, the density of surrounding tissues and possibly the age of the infected host [7]. Indeed, the yearly growth rate of cysts can be highly variable but has been suggested to be faster in younger compared to older individuals [7]. According to observations using CT, cerebral CE cysts grow on average 1 cm per year but up to 10 cm/year or 0.7 mm/month [73, 97, 98]. These observations are consistent with the reports of large CCE cysts in very young children [73, 97, 99], including on our case.

It has been suggested that CCE is more frequent in children and young adults compared to older age groups [7]. In our review, children represented over 70% of people with CCE and the mean age of CCE cases was slightly less than 20 years. While acknowledging that the real prevalence of a condition cannot be deducted from a literature review, which is limited by a number of biases (selection bias, publication bias, etc), the reasons at the basis of this apparently higher incidence of CCE in children are unknown. Some authors suggested physiological explanations based on delayed closure of the *ductus arteriosus* (ductus Botalli) [100]; however, this hypothesis is only speculative since no actual studies exist correlating occurrence of Botalli’s duct patency and extra-hepatic CE. Another hypothesis may be related, in rural endemic areas, in particular with poor hygiene conditions and habits, to the closer contact of children with contaminated environment and infected dogs. A higher probability of frequent exposures with a higher egg load in young age my cause an increase in the frequencies of CE in general and UL-CE, including CCE as well.

Studies investigating the association between CE and sex obtained contrasting results. Female sex was found a statistically significant risk factors for CE in a systematic review [101], while other large cohort study did not find any statistically significant difference between prevalence of CE in males and females [102]. Our scoping review on CCE retrieved more cases in males than females (57% *versus* 43%). Similar figures were reported by other studies on CCE, reporting a higher proportion in males [93, 97]. Again in this case, however, no strong conclusions can be drawn due to the partial and biased information that can be obtained by these studies.

### Clinical manifestations of CCE

Parasitic cysts causing CCE were documented anywhere in the brain but most frequently intra-parenchymal, rarely extradural, or a combination of these localizations [73, 97, 99]. In most clinical cases, sites most frequently involved were cerebral hemispheres in the distribution of the terminal branches of the middle cerebral artery, usually temporal-parietal-occipital [73, 97, 99]. Other sites frequently involved were the aqueduct of Sylvius, cerebellum, extradural space, diploic space of skull bones, pons, subarachnoid space, and ventricles [103].

According to what described in the literature, CCE cases presented with a wide range of clinical manifestations, which mainly depended on the localization and size of the cysts [103]. Headache, nausea and vomiting were the most common initial symptoms due to the raise of intracranial pressure. Other clinical manifestations were seizures, ataxia, hemiparesis and visual abnormalities, such as diplopia and hemianopsia [7, 9, 73, 103]. Raised intracranial pressure due to CCE, accompanied by papilledema, was also described to cause optic nerve atrophy and blindness. Mental changes, irritability and psychotic syndromes were also documented [9, 103].

### Diagnosis of CCE

Imaging plays a fundamental role in the diagnosis of CCE, as well as for all other CE localizations. In particular, CT and MRI have increased the opportunity for early diagnosis, diagnostic specificity, and have improved the outcome of the surgical interventions for CCE [9, 103]. In particular, MRI has the advantage of clearly depicting the boundaries of the cyst and its internal structures, while CT can better identify the cyst wall calcifications. On CT and MRI, unilocular fluid-filled CE cysts, classifiable as CE1 stage of the WHO-IWGE classification [104] developed for liver CE, are well-defined, thin-walled and almost always sub-spherical or sub-oval, with cyst fluid having the same density/intensity of the cerebrospinal fluid. Intact intra-parenchymal CE cysts do not show contrast enhancement and generally do not show peri-lesional edema [8]. Cyst calcifications at cerebral level were rarely documented [8, 9, 73]. Cysts with morphology classifiable in other cyst stages are encountered rarely [9], likely because cerebral cysts are usually discovered early due to appearance of symptoms cause by cyst’s growth.

Differential diagnosis of CCE cysts with other space-occupying lesions includes cysts-like masses of other parasitic origin, with different indices of suspicion depending on the epidemiological context and history (*Tania solium* causing neurocysticercosis and *Echinococcus multilocularis* causing alveolar echinococcosis), arachnoid cysts, porencephaly, cystic astrocytoma and other cystic tumors, and brain abscess [9, 105]. Due to the cyst’s very slow growth and “sealed” structure, if unruptured, absence of rim enhancement and peri-focal edema is suggestive of CCE *versus* brain abscesses and tumors [9, 73]. An increased index of suspicion should be posed for children who have lived in rural areas of endemic regions with signs of increased intracranial pressure but minimal focal deficits [106]. Moreover, a past history of abdominal or thoracic surgery for CE and recent CNS signs could increase the suspicion of CCE.

As for CE in other localizations, serology for CCE is only supportive of imaging since most of the patients with cerebral cysts often are seronegative [9]. Etiological confirmation is generally obtained after surgical interventions by histology or microscopic examination of the fluid content for the identification of protoscoleces and hooks. Molecular analyses can be implemented as confirmatory tests or for in-depth identification of species and genotypes belonging to *E*. *granulosus s*.*l*. complex. The identifications at genotype/species level of the few CCE cases (N = 20) reported in the literature for which molecular data was available cannot support any conclusion about some genotypes or species having a different tropism for the CNS (e.g. G6 genotype of *E*. *canadensis*), as hypothesized by some authors [29, 61, 92–96].

### Treatment of CCE

The treatment of CCE rely on expert opinion [107]. Surgical approaches, percutaneous cyst puncture techniques and medical treatment with albendazole were reported as the three main options applied for CCE [9]. Surgery is the treatment of choice for the complete surgical removal of the cyst without spillage of cyst content. Craniotomy and subsequent Dowling-Orlando’s technique, consisting in the instillation of hypertonic saline solution through a catheter to increase hydrostatic pressure around the cyst and evacuate it from the cavity, is the treatment of choice reported in the literature [73]. Some modifications of this surgical technique were described [108]. Puncture of intracranial cysts by percutaneous techniques such as PAIR (Puncture, Aspiration, Injection, Reaspiration), was applied for those cysts which cannot be evacuated as a whole by Dowling-Orlando’s technique, such as cysts in deep location or eloquent areas where removal of the hydatid cyst without rupturing is impossible or may cause additional neurological deficits [109]. However, the use of scolicidal agents (i.e. hypertonic saline) for CCE is not recommended since its neurotoxic effects has not been studied [9]. Standard medical treatment with albendazole (from 10 to 15 mg/kg per day) was applied for inoperable CCE cases and as a perioperative prophylaxis [104]. The use of steroids was also suggested to prevent treatment-induced perilesional edema [9].

### Outcome and follow-up of CCE

Similar to those describing other CE localizations, studies on CCE were generally lacking of long-term follow-up data, which made a robust assessment of treatment outcomes impossible. Cyst size and wall thickness were reported not to have an impact on the intact removal of cysts and surgical outcome [72]. Long-term results of the surgical treatment mainly depend on localization and number of cysts [98]. In general, single cysts, successfully removed without ruptures, had a very good outcome. In the publications included in this review, primary cysts and single cysts represented the majority (81% and 84% respectively) of documented CCE cases, thus having a potentially positive outcome. On the contrary, intraoperative rupture of cysts, multiple cysts, and difficult localizations of cysts had a poor outcome [98]. In the publications included in this review, secondary cases and multiple cysts represented a small proportion (19% and 16%, respectively) of documented CCE cases. Recurrence, permanent disability and death occurred in around 10%, 8% and 6% of cases, respectively.

Complications of CCE can be divided in preoperative, intra-operative and postoperative events. In the preoperative period, complications due to spontaneous intracranial rupture of the cysts were documented to lead to blindness, super-infection, hydrocephalus, bulging of the *dura mater*, and stroke [110]. During treatment interventions, complications were mainly caused by rupture, accidental or secondary to intentional puncture of the cyst, and anaphylactic shock in a few cases [110]. During the post-treatment period, complications were mainly related to CE recurrence but a number of other pathological manifestations were reported, such as development of a porencephalic cyst, subdural effusion, extradural hematoma, hydrocephalus, bleeding in the epidural or subdural spaces or intraparenchymal, wound infections, meningitis, abscesses, pulmonary embolism, choreiform movements, mental impairment, and seizures [103]. Clinical signs of recurrence usually appeared in a variable period between 4 and 12 months, less frequently later than 48 months, after treatment. In the recurrent cases, clinical course was complicated with poor prognosis due to multiple intracranial cysts [8].

## Conclusions

We took from this report on a case of primary CCE in a child who was born and lived at the outskirts of Rome to explore the possible origin of infection by means of comparative molecular approach and to summarize the characteristic of cases with this uncommon localization reported in the literature. A comparative molecular analysis with specimens of *E*. *granulosus s*.*l*. from sheep from both the farm of the child’s family and from one neighbouring farm, as well as reference strains, identified that the species involved was *E*. *granulosus s*.*s*. (G3 genotype) and supported the view that the infection was probably acquired in the household/backyard of the family farm. While we could not carry out an analysis of all potentials sources of infection (that is, matrices such as farms’ dogs feces, farm’s soil, etc.), far as we know, this is the first time source attribution of human infection at local scale was attempted.

Case-series analysed in this review confirmed figures commonly reported that liver and lung localizations (around 70% and 20%, respectively) are the most common localizations of CE in clinical cohorts, while UL-CE represent around 10%, with 1.5% of all documented CE cases having cerebral localisations. Based on recently published estimates of about 65,000 CE cases in Europe during the period 1997–2021 [111], we can roughly estimate that 10% of them (N = 6,500) could be due to UL-CE, of which around 975 cases of CCE. These estimates should help better defining the burden of CE when considering these highly disabling cases, which might be underestimated when compared to burden calculations carried out for AE [112]. Indeed, even if global estimates on the number of yearly incidence of AE is around 1/10 of that of CE (approximately 18,000 *versus* 200,000 cases), AE has higher DALYs compared to CE [112, 113]. This is due to the higher mortality rate of AE, which in turn has a greater weight in the DALY health metric. However, UL-CE cases are often clinically complex to manage with a devastating impact on humans (in particular osseous, cerebral, spinal and cardiac CE). Our rough estimates on number of UL-CE cases equal the number of AE cases globally. If the proportion of UL-CE cases within CE global estimates are taken into account, the calculated magnitude of the burden of CE would change. This suggests that human CE burden may have been globally neglected, in particular when comparing with other foodborne parasitic diseases [112].

## Supporting information

S1 Fig Preferred Reporting Items for Systematic reviews and Meta-Analyses extension for Scoping Reviews (PRISMA-ScR) Checklist. (PDF)

S2 Fig Literature search flow diagram. (TIF)

S1 Text List of publications from which data where extracted. (PDF)
