# Supplementary figures and images for: Primary cerebral cystic echinococcosis in a child from Roman countryside: Source attribution and scoping review of cases from the literature
Source: PLoS Negl Trop Dis. 2023 Sep 5;17(9):e0011612. doi: 10.1371/journal.pntd.0011612 (PMC10503711; doi:10.1371/journal.pntd.0011612)

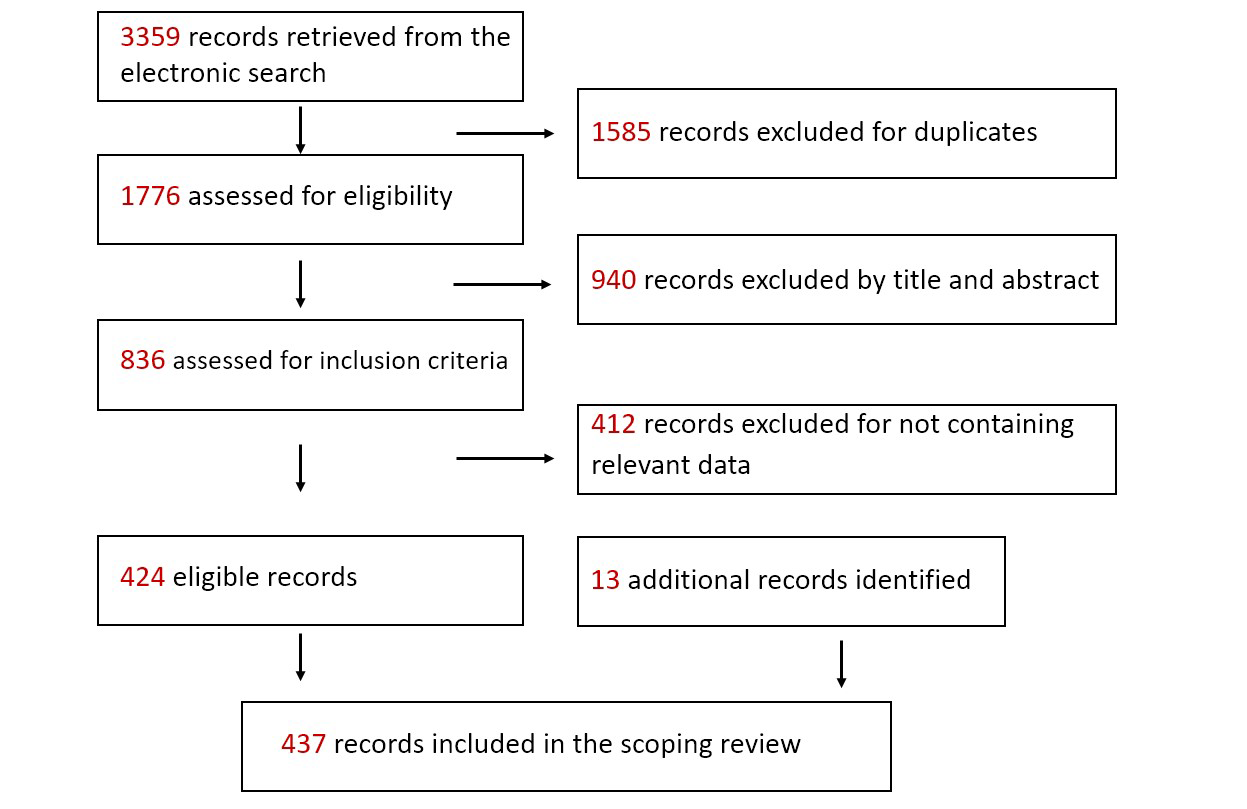

Supplement: S2 Fig — (TIF) [file pntd.0011612.s002.tif]
