# Supplementary material for: Primary cerebral cystic echinococcosis in a child from Roman countryside: Source attribution and scoping review of cases from the literature
Source: PLoS Negl Trop Dis. 2023 Sep 5;17(9):e0011612. doi: 10.1371/journal.pntd.0011612 (PMC10503711; doi:10.1371/journal.pntd.0011612)
Supplement: S1 Text — (PDF) [file pntd.0011612.s003.pdf]

LIST OF PUBLICATIONS FROM WHICH DATA WHERE EXTRACTED

1. Andreula C, Vreto G, Santini E, Podda P, Osmanlli A, Alimehmeti R, Petrela M. Extradural intracranial hydatid cyst with pathognomonic “Nail Scratch” sign on the theca interna: A Case Report. Rivista di Neuroradiologia. 2000;13(5):749-754.
2. Abbassioun K, Amirjamshidi A, Moinipoor MT. Hydatid cyst of the pons. Surg Neurol. 1986;26(3):297-300.
3. Abbassioun K, Rahmat H, Ameli NO, Tafazoli M. Computerized tomography in hydatid cyst of the brain. J Neurosurg. 1978;49(3):408-11.
4. Abbassioun K. Intracerebral hydatid cyst; a review of 22 cases. IJMS-Iranian Journal of Medical Sciences. 1986;13(1):2-9.
5. Abdel Razek AA, El-Shamam O, Abdel Wahab N. Magnetic resonance appearance of cerebral cystic echinococcosis: World Health Organization (WHO) classification. Acta Radiol. 2009;50(5):549-54.
6. Abdulla K, Tapoo AK, Agha HS. Ruptured cerebral hydatid cyst: a case report. J Trop Med Hyg. 1988 Dec;91(6):302-5.
7. Abu-Eshy SA. Some rare presentations of hydatid cyst (*Echinococcus granulosus*). J R Coll Surg Edinb. 1998;43(5):347-52.
8. Ahadi R, Rostamzadeh A, Naleini F, and Fatehi D. Cerebral Echinococcosis. Zahedan J Res Med Sci. 2015; 17(11):e2202.
9. Ahmadi NA, Badi F. Human hydatidosis in Tehran, Iran: a retrospective epidemiological study of surgical cases between 1999 and 2009 at two university medical centers. Trop Biomed. 2011 Aug;28(2):450-6.
10. Ahmadi NA, Hamidi M. Unusual localizations of human hydatid disease in Hamedan province, west of Iran. Helminthologia. 2010;47(2):94-98.
11. Akbar A. Patterns of intracranial mass lesions - Experience of 78 cases. Journal of the College of Physicians and Surgeons Pakistan. 2001;11(9):556-8.
12. Akcam AT, Ulku A, Koltas IS, Izol V, Bicer OS, Kilicbagir E, Sakman G, Poyrazoglu H, Erman T, Aridogan IA, Parsak CK, Inal M, Iskit S. Clinical characterization of unusual cystic echinococcosis in southern part of Turkey. Ann Saudi Med. 2014;34(6):508-16.
13. Akdemir G, Dağlioğlu E, Seçer M, Ergüngör MF. Hydatid cysts of the internal acoustic canal and jugular foramen. J Clin Neurosci. 2007;14(4):394-6.
14. Akdemir G, Ergün R, Gezici AR, Ökten AI, Ergüngör MF. Multiple hydatid cysts of the aqueduct of sylvius: a case report with MRI study. Turkish Neurosiirgery 2000;10:142-144.
15. Akkaya H, Akkaya B, Gönülcü S. Hydatid disease involving some rare sites in the body. Turkiye Parazitol Derg. 2015;39(1):78-82.
16. Akrim Y, Barkate K, Arrad Y, Ghannane H, El Hakkouni A. Multiple Cerebral Hydatid Cysts: A Case Report. Cureus. 2022;31;14(5):e25529.
17. Al Zain TJ, Al-Witry SH, Khalili HM, Aboud SH, Al Zain FT Jr. Multiple intracranial hydatidosis. Acta Neurochir (Wien). 2002;144(11):1179-85.
18. Al-Akayleh, A. Intracranial hydatid cysts: diagnosis and treatment. Bahrain Med Bull. 2003;25:91-4.
19. Alam S, Umer US, Gul S, Ghaus S, Farooq B, Gul F. Uncommon sites of a common disease - Hydatid cyst. J Postgrad Med Inst 2014;28(3):270-6.
20. al-Bassam A, Hassab H, al-Olayet Y, Shadi M, al-Shami G, al-Rabeeah A, Jawad A. Hydatid disease of the liver in children. Ann Trop Paediatr. 1999;19(2):191-6.
21. Aleksic-Shihabi A, Vidolin EP. Cystic echinococcosis of the heart and brain: a case report. Acta Med Okayama. 2008;62(5):341-4.
22. Ali M, Mahmood K, Khan P. Hydatid cysts of the brain. J Ayub Med Coll Abbottabad. 2009;21(3):152-4.
23. Alkhotani A, Butt B, Khalid M, Binmahfoodh M. Peripontomedullary hydatid cyst: Case report and literature review. Int J Surg Case Rep. 2019;55:23-27.
24. Alok R, Mahmoud J. Successful Surgical Treatment of a Brain Stem Hydatid Cyst in a Child. Case Rep Surg. 2020 23;2020:5645812.
25. Alomari MS, Almutairi MK, Alali HM, Elwir JS, Alola SA, Alfattoh NI, Alharthy NA, Azzubi MA. Primary Giant Cerebral Hydatid Cyst in an 8-year-old Girl. Asian J Neurosurg. 2018;13(3):800-802.
26. Al-Raw WW. Cerebral hydatid disease patients admitted to Duhok city hospitals. Proceedings of the 2016 Spring Meeting of the Society of British. British Journal of Neurosurgery. 2016;30(2):130-186.

27. Altas M, Serarslan Y, Davran R, Evirgen O, Aras M, Yilmaz N. The Dowling-Orlando technique in a giant primary cerebral hydatid cyst: a case report. Neurol Neurochir Pol. 2010;44(3):304-7.
28. Altibi AM, Qarajeh RA, Belsuzarri TA, Maani W, Kanaan TM. Primary cerebral echinoccocosis in a child: Case report - Surgical technique, technical pitfalls, and video atlas. Surg Neurol Int. 2016;21;7(37):S893-S898.
29. Altinörs N, Bavbek M, Caner HH, Erdogan B. Central nervous system hydatidosis in Turkey: a cooperative study and literature survey analysis of 458 cases. J Neurosurg. 2000;93(1):1-8.
30. Altinörs N, Senveli E, Dönmez T, Bavbek M, Kars Z, Sanli M. Management of problematic intracranial hydatid cysts. Infection. 1995;23(5):283-7.
31. Altıntaş Taşlıçay C, Dervişoğlu E, Babaoglu A, Anık Y. Co-occurrence of cardiac hydatid cyst with cerebral embolisation and vascular hydatid cyst. J Paediatr Child Health. 2019;55(11):1391-1394.
32. Alvarez F, Blazquez MG, Oliver B, Manrique M. Calcified cerebral hydatid cyst. Surg Neurol. 1982;17(3):163-4.
33. Ameen AA. Brain hydatid cyst in Basrah. Trop Doct. 1986;16(2):63-5.
34. Amin OS. Multiple cerebral hydatid cysts: have the previous operations contributed to their formation? BMJ Case Rep. 2012 Oct 6;2012:bcr2012007240.
35. Amir-Jahed AK, Fardin R, Farzad A, Bakshandeh K. Clinical echinococcosis. Ann Surg. 1975;182(5):541-6.
36. Amr SS, Amr ZS, Jitawi S, Annab H. Hydatidosis in Jordan: an epidemiological study of 306 cases. Ann Trop Med Parasitol. 1994;88(6):623-7.
37. Anderson M, Bickerstaff ER, Hamilton JG. Cerebral hydatid disease in Britain. J Neurol Neurosurg Psychiatry. 1975;38(11):1104-8.
38. Andronikou S, Welman CJ, Kader E. Classic and unusual appearances of hydatid disease in children. Pediatr Radiol. 2002;32(11):817-28.
39. Anvari M, Amirjamshidi A, Abbassioun K. Gradual and complete delivery of a hydatid cyst of the brain through a single burr hole, a wrong happening! Childs Nerv Syst. 2009;25(12):1639-42.
40. Arana-Iñiguez R, & San Julián J. Hydatid cysts of the brain. Journal of Neurosurgery. 1955;12(4):323-35.
41. Arcidiacono G, Di Mauro C, Zingali C, Gurgone G, Asmundo GO, Borzì S, Cuscunà S, Garozzo G, Longhitano A, Mammana A, Mughini L. Echinococcosi cerebrale primaria solitaria. Descrizione di un caso [Solitary primary cerebral echinococcosis. Minerva Med. 1997;88(3):87-92.
42. Arora SK, Aggarwal A, Datta V. Giant primary cerebral hydatid cyst: A rare cause of childhood seizure. J Pediatr Neurosci. 2014;9(1):73-5.
43. Arseni C, Marinescu V. Epilepsy in cerebral hydatidosis. Epilepsia. 1974;15(1):45-54.
44. Arseni C, Samitca DC. Cranial and cerebral hydatid disease. Acta Psychiatr Neurol Scand. 1957;32(4):389-98.
45. Ashraf M, Ahmed S, Ahmad S, Ahmad A. A Large Hydatid Cyst in the Brain of a 10-year Child. J Coll Physicians Surg Pak. 2022;32(4):538-540.
46. Assamadi M, Benantar L, Hamadi H, Ksiks O, El Hadwe S, Aniba K. Cerebral hydatid cyst in children: A case series of 21 patients and review of literature. Neurochirurgie. 2022;68(6):618-626.
47. Assefa G, Biluts H, Abebe M, Birahanu MH. Cerebral hydatidosis, a rare clinical entity in Ethiopian teaching hospitals: case series and literature review. East and Central African Journal of Surgery. 2011;16(2):123-129.
48. Atalar MH, Arslan M, Petik B. Cerebral hydatid cysts in childhood: Computed tomography findings - Case reports. Turkiye Klinikleri Journal of Medical Sciences. 2005;25(2):313-315.
49. Aydin MD, Aydin N. A Cerebral Hydatid Cyst Case First Presenting with Gerstmann's Syndrome: A Case Report and Literature Review. Turkish Journal of Medical Sciences. 2003;33(1):57-60.
50. Aydin MD, Karaavci NC, Akyuz ME, Sahin MH, Zeynal M, Kanat A, Altinors MN. A New Technique in Surgical Management of the Giant Cerebral Hydatid Cysts. J Craniofac Surg. 2018;29(3):778-782.
51. Aydin MD, Ozkan U, Altinörs N. Quadruplets hydatid cysts in brain ventricles: a case report. Clin Neurol Neurosurg. 2002;104(4):300-2.
52. Aydin Y, Aydin F, Ture U. Intradiploeic and cerebral hydatidosis: a case report and review of literature. Clin Neurol Neurosurg. 1992;94(3):229-33.
53. Ayres CM, Davey LM, German WJ. Cerebral hydatidosis. Clinical case report with a review of pathogenesis. J Neurosurg. 1963;20:371-7.
54. Baboli S, Baboli S, Soleiman Meigooni S. Brain Hydatid Cyst with Atypical Symptoms in an Adult: A Case Report. Iran J Parasitol. 2016;11(3):422-425.
55. Bahloul K, Ouerchefani N, Kammoun B, Boudouara MZ. Unusual brain edema caused by an intracranial hydatid cyst: case report and literature review. Neurochirurgie. 2009;55(1):53-6.

56. Bakaris S, Sahin S, Yuksel M, Karabiber H. A large cerebral hydatid cyst associated with liver cyst. Ann Trop Paediatr. 2003;23(4):313-7.
57. Bakhsh A, Siddiqui KMA, Taraif S. Primary hydatid cyst of pineal region of brain: A case report from Saudi Arabia. Asian J Neurosurg. 2017;12(2):314-317.
58. Balak N, Bayindir C, Uzuner E. Do cyst wall thickness and cyst size have any effect on the intra-operative inadvertent rupture of echinococcal hydatid cyst of central nervous system? Clin Neuropathol. 2009;28(3):203-9.
59. Balak N, Cavumirza C, Yildirim H, Ozdemir S, Kinay D. Microsurgery in the removal of a large cerebral hydatid cyst: technical case report. Neurosurgery. 2006;59(4 Suppl 2):ONSE486.
60. Banzo J, Diaz FJ, Pina JI, Abós MD, Rios G, Garcia D, Marín F. Multiple cerebral hydatid cysts. Eur J Nucl Med. 1984;9(12):561-3.
61. Baradan Bagheri A, Zibaei M, Tayebi Arasteh M. Cystic Echinococcosis: A Rare Case of Brain Localization. Iran J Parasitol. 2017;12(1):152-155.
62. Bartosch C, Reis C, Castro L. Large solitary cerebral hydatid cyst. Arch Neurol. 2011;68(7):946-7.
63. Baruah A, Sarma K, Barman B, Phukan P, Nath C, Boruah P, Rajkhowa P, Baruah M, Dutta A, Naku N. Clinical and laboratory presentation of hydatid disease: A study from northeast India. Cureus. 2020; 5;12(9):e10260.
64. Basarslan SK, Gocmez C, Kamasak K, Ceviz A. The Gigant primary cerebral hydatid cyst with no marked manifestation: a case report and review of literature. Eur Rev Med Pharmacol Sci. 2015;19(8):1327-9.
65. Baysefer A, Erdoğan E, Gönül E, Kayali H, Tímurkaynak E, Seber N. Primary multiple cerebral hydatid cysts: case report with CT and MRI study. Minim Invasive Neurosurg. 1998;41(1):35-7.
66. Begg NC, Begg AC, Robinson RG. Primary hydatid disease of the brain: its diagnosis, radiological investigation, treatment and prevention. N Z Med J. 1957;56(312):84-98.
67. Behari S, Banerji D, Phadke RV, Shukla S, Krishnani N, Chhabra DK. Multiple infected extradural parasellar hydatid cysts. Surg Neurol. 1997;48(1):53-7.
68. Belahcen M, Khattala K, Elmadi A, Bouabdellah Y. Kyste hydatique cérébral chez l'enfant: à propos de 5 cas [Cerebral hydatid cyst in children: report of 5 cases]. Pan Afr Med J. 2014, 3;17:149.
69. Belfquih H, Azami MA, Akhaddar A. Rare Case of Hydatid Cyst in the Cerebellopontine Cistern. World Neurosurg. 2021;145:48-50.
70. Benomar A, Yahyaoui M, Birouk N, Vidailhet M, Chkili T. Middle cerebral artery occlusion due to hydatid cysts of myocardial and intraventricular cavity cardiac origin. Two cases. Stroke. 1994;25(4):886-8.
71. Benzagmout M, Maaroufi M, Chakour K, Chaoui ME. Atypical radiological findings in cerebral hydatid disease. Neurosciences (Riyadh). 2011;16(3):263-6.
72. Beşkonakli E, Cayli S, Yalçinlar Y. Primary intracranial extradural hydatid cyst extending above and below the tentorium. Br J Neurosurg. 1996;10(3):315-6.
73. Beskonakli E, Solaroglu I, Tun K, Albayrak L. Primary intracranial hydatid cyst in the interpeduncular cistern. Acta Neurochir (Wien). 2005;147(7):781-3.
74. Bhaskar S, Choudhary A, Singh AK. Posterior fossa extradural hydatid cyst with septae and calcification. Neurol India. 2012;60(2):242-3.
75. Bhatt AS, Mhatre R, Nadeesh BN, Mahadevan A, Yasha TC, Santosh V. Nonneoplastic Cystic Lesions of the Central Nervous System-Histomorphological Spectrum: A Study of 538 Cases. J Neurosci Rural Pract. 2019;10(3):494-501.
76. Bilge T, Barut S, Bilge S, Aydin Y, Aksoy B, Senol S. Primary multiple hydatid cysts of the brain: case report. Surg Neurol. 1993;39(5):377-9.
77. Binesh F, Mehrabanian M, Navabii H. Primary brain hydatosis. BMJ Case Rep. 2011 Mar 8;2011:bcr0620103099.
78. Bleil CB, Soares FP, Velho MC, Roncaglio R, Vione MC. Intracranial echinococcosis in pediatric patient-Case report and illustrative video. Childs Nerv Syst. 2013;29(9):1757.
79. Boles DM. Cerebral echinococciasis. Surg Neurol. 1981;16(4):280-2.
80. Bora G, Sünnetçioğlu A, Sünnetçioğlu M, Bora A, Yılmaz Ö, Çim N, Baran Aİ. Examination of atypical located hydatid cyst disease in Eastern Turkey. Eastern J Med. 2016;21(4):158-161.
81. Borkar SA, Verma N, Joseph SL, Kale SS, Mahapatra AK. Magnetic Resonance Imaging of Pediatric Primary Cerebral Hydatidosis. J Pediatr Neurosci. 2017;12(3):298-299.
82. Bottieau E, David P, Dewitte O, Jacobs F. Eosinophilic meningitis following incomplete resection of a meningeal hydatid cyst. Scand J Infect Dis. 2003;35(11-12):898-901.

83. Bougharriou I, Elleuch E, Ben Hmida S, Meddeb A, Boudaouara Z, Kammoun B, Ben Jemaa M. Cystic lesions of the brain: Think of the hydatid cyst. Tunis Med. 2022;100(1):56-59.
84. Bouguerra L. Brain hydatidosis diagnosed by computed tomography. J Trop Pediatr. 1990;36(5):268-9.
85. Boujan MM, Jalal ZS. A rare case of primary multiple hydatid cysts of the brain in a 10-year-old child. East Mediterr Health J. 2014;9;19(3):S216-9.
86. Bozdoğan, D, Eyileten, Z, Aliyev, A, Demir, F, Tutar, E, Atalay, S, Uysalel, A. PP-417 Cardiac involvement of multivisseral hydatidosis in a child. International Journal of Cardiology. 2012;(155):S224.
87. Braham E, Bellil S, Bellil K, Chelly I, Mekni A, Haouet S, Kchir N, Khaldi M, Zitouna M. Kyste hydatique de la fosse postérieure [Hydatid cyst of the posterior fossa]. Med Mal Infect. 2007;37(5):281-3.
88. Braunsdorf EW, Schmidt D, Rautenberg M. Cerebral manifestation of hydatid disease in a child. Childs Nerv Syst. 1988;4(4):249-51.
89. Brook P, Hill B and Saad N. Asian Oceanian Congress of Radiology 2012 and The Royal Australian and New Zealand College of Radiologists 63rd Annual Scientific Meeting. Journal of Medical Imaging and Radiation Oncology. 2012;56:163-225.
90. Bull JW, Schunk H. The significance of displacement of the cavernous portion of the internal carotid artery. Br J Radiol. 1962;35:801-14.
91. Busić Z, Bradarić N, Ledenko V, Pavlek G. Cystic echinococcosis of lung and heart coupled with repeated echinococcosis of brain-a case report. Coll Antropol. 2011;35(4):1311-5.
92. Çakir M, Çalikoglu Ç, Yilmaz A. A Very Rare Complication of Cerebral Hydatid Cyst Surgery: Cortical Collapse. J Pediatr Neurosci. 2017;12(4):346-348.
93. Cam İ, Çakır Ö, Tekin Yılmaz A, Genez S, Anık Y. Unusual Presentation of Multi-organ Hydatid Cysts in a Child. Balkan Med J. 2019;22;36(5):292-293.
94. Canbolat A, Onal C, Kaya U, Coban TE. Intracranial extradural hydatid cysts: report of three cases. Surg Neurol. 1994;41(3):230-4.
95. Carrea R, Dowling E Jr, Guevara JA. Surgical treatment of hydatid cysts of the central nervous system in the pediatric age (Dowling's technique). Childs Brain. 1975;1(1):4-21.
96. Carroll JD, Lascelles RG. Hydatid disease in the posterior fossa. Br Med J. 1962;28;2(5299):234-6.
97. Cataltepe O, Colak A, Ozcan OE, Ozgen T, Erbengi A. Intracranial hydatid cysts: experience with surgical treatment in 120 patients. Neurochirurgia (Stuttg). 1992;35(4):108-11.
98. Çataltepe O, Tahta K, Çolak A, Erbengi A. Primary multiple cerebral hydatid cysts. Neurosurg Rev. 1991;14(3):231-4.
99. Çavuş G, Açik V, Çavuş Y, Bilgin E, Gezercan Y, Ökten AI. An extraaxially localized intrasellar giant hydatid cyst with hypophyseal insufficiency. Childs Nerv Syst. 2018;34(7):1391-1396.
100. Cavuşoğlu H, Tuncer C, Ozdilmaç A, Aydin Y. Multiple intracranial hydatid cysts in a boy. Turk Neurosurg. 2009;19(2):203-7.
101. Cece H, Sogut O, Kaya H. Primary giant intracranial extracerebral hydatid disease in a child. J Pak Med Assoc. 2011;61(8):826-7.
102. Çelebi S, Bozdemir ŞE, Taşkapılıoğlu Ö, Hacımustafaoğlu M. Primary Solitary and Multiple Intracranial Hydatid Cyst Disease: Report of Four Cases. J Pediatr Inf 2018;12(3):e110-e114.
103. Cemil B, Tun K, Gurcay AG, Uygur A, Kaptanoglu E. Cranial epidural hydatid cysts: clinical report and review of the literature. Acta Neurochir (Wien). 2009;151(6):659-62.
104. Chand K, Kanodia AK, Manpreet G, Neeraj A. In vivo proton magnetic resonance spectroscopy in a known case of intracranial hydatid cyst. Neurol India. 2005;53(3):337-8.
105. Chatzidakis E, Zogopoulos P, Paleologos TS, Papageorgiou N. Surgical Planning for the Treatment of a Patient with Multiple, Secondary, Intracranial Echinococcal Cysts. Surg J (N Y). 2015;18;2(1):e7-e10.
106. Chaurasia AS, Nawale JM, Patil SN, Yemul MA, Mukhedkar S, Sharma SK, K V, Punjabi P. Cystic hydatidosis of the heart and brain. Echocardiography. 2012;29(8):E208-9.
107. Chen J, Wu X, He Y, Li S, Deng Y, Chen J, Fang W, Zeren Z, Peng J, Li Y, Mu J, Zhou D. A Retrospective Analysis of the Clinical Features of Inpatients With Epilepsy in the Ganzi Tibetan Autonomous Prefecture. Front Neurol. 2018;30;9:891.
108. Chen S, Li N, Yang F, Wu J, i Hu Y, Yu S, Chen Q, Wang X, Wang X, Liu Y & Zheng J. Medical treatment of an unusual cerebral hydatid disease. BMC Infectious Diseases. 2018;5;18(1):12.

109. Ciurea AV, Fountas KN, Coman TC, Machinis TG, Kapsalaki EZ, Fezoulidis NI, Robinson JS. Long-term surgical outcome in patients with intracranial hydatid cyst. Acta Neurochir (Wien). 2006;148(4):421-6.
110. Ciurea AV, Vasilescu G, Nuteanu L, Carp N. Cerebral hydatid cyst in children. Experience of 27 cases. Childs Nerv Syst. 1995;11(12):679-85.
111. Clements R, Gravelle IH. Radiological appearances of hydatid disease in Wales. Postgrad Med J. 1986;62(725):167-73.
112. Coates R, von Sinner W, Rahm B. MR imaging of an intracerebral hydatid cyst. AJNR Am J Neuroradiol. 1990;11(6):1249-50.
113. Cohen JE, Ginsberg HJ, Tsai EC, Schwartz ML, Petrocelli S. Hydatid cyst and brain tumor in the same location. Case illustration. J Neurosurg. 1997;86(2):312.
114. Cöl C, Cöl M, Lafçi H. Unusual localizations of hydatid disease. Acta Med Austriaca. 2003;30(2):61-4.
115. Copley IB, Fripp PJ, Erasmus AM, Otto DD. Unusual presentations of cerebral hydatid disease in children. Br J Neurosurg. 1992;6(3):203-10.
116. Costa-Pinho A, Oliveira MA, Graça LA, Costa-Maia J. Cerebral and hepatic hydatid disease. Porto Biomed J. 2016;1(4):153-154.
117. Dagtekin A, Koseoglu A, Kara E, Karabag H, Avci E, Torun F, Bagdatoglu C. Unusual location of hydatid cysts in pediatric patients. Pediatr Neurosurg. 2009;45(5):379-83.
118. Darwazah AK, Zaghari M, Eida M, Batrawy M. Left ventricular endocardial ecchinococcosis associated with multiple intracranial hydatid cysts. J Cardiothorac Surg. 2013;8:104.
119. Daskas N, Aggelopoulos E, Tzoufi M, Kosta P, Siamopoulou A, Argyropoulou MI. Accidental drainage of a cerebral hydatid cyst into the peritoneal cavity. Pediatr Infect Dis J. 2004;23(7):685-6.
120. Dastur DK, Lalitha VS. Pathological analysis of intracranial space-occupying lesions in 1000 cases including children. 4. Pituitary adenomas; developmental tumours; parasitic and developmental cysts. J Neurol Sci. 1972;15(4):397-427.
121. De Villers Hamman H, Joubert MJ. A review of hydatid disease of the brain. S Afr Med J. 1957;16;31(46):1163-7.
122. Demir K, Karsli AF, Kaya T, Devrimci E, Alkan K. Cerebral hydatid cysts: CT findings. Neuroradiology. 1991;33(1):22-4.
123. Demir MK, Yapıcıer O, Hasanov T, Yılmaz B, Kılıç T. Hydatid disease: MR imaging of calvarium and superior sagittal sinus involvement. Neuroradiol J. 2018;31(3):320-323.
124. Demir MK, Yapıcıer Ö, Jameel MA, Bozbuğa M. Cerebral hydatid disease with serpent sign, calcifications, and peripheral enhancement. Acta Neurol Belg. 2020;120(5):1173-1175.
125. Devi Indira B. Cerebellopontine angle extradural hydatid cyst mimicking an intracranial abscess. Indian J. Otol. 2004;10:41-4.
126. Dew HR. Primary cerebral hydatid disease. Aust N Z J Surg. 1955;24(3):161-71.
127. Dharker SR, Dharker RS, Vaishya ND, Sharma ML, Chaurasia BD. Cerebral hydatid cysts in central India. Surg Neurol. 1977;8(1):31-4.
128. Dhingra D, Sethi GR, Mantan M. Intracranial hydatid cyst. Indian J Pediatr. 2014;81(2):212.
129. Diaz de Durana MD, Lopez A, Fraj J. Anaphylaxis and cerebral hyadatidic disease. Ann Intern Med. 1997;1;126(9):745.
130. Díaz P, Maillo A. Oclusión de la arteria cerebral media tras la embolización de material parasitario hidatídico en una paciente en la edad pediátrica: caso clínico [Middle cerebral artery occlusion after cerebral hydatic embolism in a pediatric patient: case report]. Neurocirugia (Astur). 2002;13(3):216-8.
131. Diren HB, Ozcanli H, Boluk M, Kilic C. Unilocular orbital, cerebral and intraventricular hydatid cysts: CT diagnosis. Neuroradiology. 1993;35(2):149-50.
132. Duishanbai S, Geng D, Liu C, Guo HR, Hao YJ, Liu B, Wang YX, Luo K, Zhou K, Wen H; Research Group of Hydatid Diseases. Treatment of intracranial hydatid cysts. Chin Med J (Engl). 2011;124(18):2954-8.
133. Duishanbai S, Jiafu D, Guo H, Liu C, Liu B, Aishalong M, Mijiti M, Wen H. Intracranial hydatid cyst in children: report of 30 cases. Childs Nerv Syst. 2010;26(6):821-7.
134. Ekici MA, Ekici A, Per H, Tucer B, Kumandaş S, Kurtsoy A. Concomitant heart and brain hydatid cyst without other organ involvement: a case report / Diğer organların tutulumu olmaksızın eş zamanlı kalp ve beynin kist hidatik tutulumu: Olgu bildirimi. Dusunen Adam: The Journal of Psychiatry and Neurological Sciences. 2011;155.159.
135. El Ouarradi A, Oualim S, Bensahi I, Elkouhen M, Abouloiafa I, Sabry M. Brain and Cardiac Concomitant Localization of the Hydatid Cyst. Case Rep Pediatr. 2020;18;2020:4829496.

136. Elamour S, Ben-Shimol S, Melamed I. Hydatid Brain Cyst in a Limping Child. Am J Trop Med Hyg. 2022;14;107(6):1155-1156.
137. El-Shamam O, Amer T, El-Atta MA. Magnetic resonance imaging of simple and infected hydatid cysts of the brain. Magn Reson Imaging. 2001;19(7):965-74.
138. Elvan-Tuz A, Karadag-Oncel E, Kara-Aksay A, Sarioglu FC, Karadag A, Yilmaz-Ciftdogan D. A rare case series of central nervous system cystic echinococcosis. J Trop Pediatr. 2021;2;67(3):fmab056.
139. Emamy H, Bcheshti GH, Shokoohi SH, Radjaieh SH, Mosavy SH. Multi hydatid of parotid, brain and kidney. Report of a case. Iranian Journal of Public Health. 1974;3(4):201-5.
140. Erdinçler P, Kaynar MY, Babuna O, Canbaz B. The role of mebendazole in the surgical treatment of central nervous system hydatid disease. Br J Neurosurg. 1997;11(2):116-20.
141. Erkutlu I, Buyukhatipoglu H, Alptekin M, Gok A. Where is the brain paranchyme? "Primary cerebral multiple cyst hydatid". Intern Med. 2008;47(4):333.
142. Erman T, Tuna M, Göçer I, Ildan F, Zeren M, Cetinalp E. Intracranial intraosseous hydatid cyst. Case report and review of literature. Neurosurg Focus. 2001;15;11(1):ECP1.
143. Erongun U, Ozkal E, Acar O, Kocaoğullar Y. Multiple and infected cerebral hydatid cysts. Case report. Neurosurg Rev. 1994;17(1):77-81.
144. Erşahin Y, Mutluer S, Dermirtaş E, Yurtseven T. A case of thalamic hydatid cyst. Clin Neurol Neurosurg. 1995;97(4):321-3.
145. Eser I, Karabag H, Gunay S, Seker A, Cevik M, Ali Sak ZH, Yalcin F, Aydin MS. Surgical approach for patients with unusually located hydatid cyst. Ann Ital Chir. 2014;85(1):50-5.
146. Etaiwi AB. Lift suboccipital retrosegmoid craniectomy for removal of lift cerebellar hydatid cyst & treatment of hydrocephalus with out CSF shunting. A case report. Childs Nerv Syst. 2015;31(10):1988.
147. Evliyaoglu C, Yuksel M, Gul B, Kaptanoglu E, Yaman M. Growth rate of multiple intracranial hydatid cysts assessed by CT from the time of embolisation. Neuroradiology. 1998;40(6):387-9.
148. Fabiani A, Trebini F, Torta R. Brain hydatidosis: report of two cases. J Neurol Neurosurg Psychiatry. 1980;43(1):91-4.
149. Fakhouri F, Ghajar A, Mahli N, Shoumal N. Giant hydatid cyst in the posterior fossa of a child. Asian J Neurosurg. 2015;10(4):322-4.
150. Farajirad E, Farajirad M, Khajavi M, Shojaie SRH. Central Nervous System Hydatid Disease: Clinical Analysis of 99 Cases in Qaem Hospital of Mashad University of Medical Sciences, Iran. Neurosurgery Quarterly. 2016;26(1):1-4.
151. Fares Y, El-Zaatari M, Haddad G, Kanj A. Cerebral hydatid cyst: Successfully managed. Pan Arab Journal of Neurosurgery 2011;15(2):9-11.
152. Fateh S, Mohammadi A, Beyranvand M. A Case of Hydatid Cyst Recurrence in Different Areas of the Body. Journal of Arak University of Medical Sciences. 2017;20(4):40-5.
153. Fleta J, Sarria A, Villagrasa J, Usón A, Ramos FJ, Bueno M, Calatayud V. Computed tomography in the diagnosis of brain hydatidosis in children. Acta Paediatr Scand. 1987;76(5):835-6.
154. Foster PS. Hydatid cyst of the brain. Aust N Z J Surg. 1949;18(3):228.
155. Furtado SV, Visvanathan K, Nandita G, Reddy K, Hegde AS. Multiple fourth ventricular hydatidosis. J Clin Neurosci. 2009;16(1):110-2.
156. Fuster B, Castells C, GastauT H. The electroencephalographic study of hydatid cysts of the brain. Electroencephalogr Clin Neurophysiol. 1955;7(3):415-20.
157. Gana R, Skhissi M, Maaqili R, Bellakhdar F. Multiple infected cerebral hydatid cysts. J Clin Neurosci. 2008;15(5):591-3.
158. García-Uria J, Cabezudo J, Nombela L. Subdural haematoma as a complication in the surgical removal of intracranial hydatidosis. Acta Neurochir (Wien). 1980;52(1-2):51-4.
159. Garg M, Sarma P, Chaturvedi S, Pant I. Multiple Primary Bilateral Cerebral Echinococcosis in an Adult: A Neurological Rarity. Asian J Neurosurg. 2022;18;17(4):647-650.
160. Gargouri L, Aoud I El, Hammemi F, Regaieg C, Safi F, Gargouri A, Mnif Z, Jemaa MB, Koubaa M, Mahfoudh A. P621 Hydatidosis in infants: what not to be missed. Archives of Disease in Childhood. 2019;104:A398-A399.
161. Gautam S, Sharma A. Intracranial Hydatid Cyst: A Report of Three Cases in North-West India. J Pediatr Neurosci. 2018;13(1):91-95.
162. Geiger LE. Hydatid cyst of the brain. Report of a case. J Neurosurg. 1965;23(4):446-9.

163. Gezen F, Baysefer A, Köksel T, Gönül E, Akay KM, Erdogan E. Hydatid cysts of the brain. Clin Infect Dis. 1995;21(4):938-42.
164. Ghasemi, Amir A. Hydatid Cyst of the Brain. Neurosurgery Quarterly. 2014 24(2):136-138.
165. Gökalp HZ, Erdoğan A. Hydatid cyst of the aqueduct of Sylvius. Case report. Clin Neurol Neurosurg. 1988;90(1):83-5.
166. Gökalp HZ, Kanpolat Y, Gökben B. Hydatid cyst of the skull. Neurosurgery. 1979;4(5):431-3.
167. Gomori JM, Cohen D, Eyd A, Pomeranz S. Water lily sign in CT of cerebral hydatid disease: a case report. Neuroradiology. 1988;30(4):358.
168. González-Ruiz CA, Isla A, Pérez-Higueras A, Blázquez MG. Unusual CT image of a cerebral hydatid cyst. Pediatr Radiol. 1990;20(4):283-4.
169. Gordillo I, Millán JM, Escudero L, Orduna M, Roger R, de la Fuente M. Multiple intracranial hydatid cysts. Neuroradiology. 1986;28(3):285.
170. Guler I, Nayman A, Erdogan H, Koplay M, Paksoy Y. Cranial hydatid cyst initially presented with restricted diffusion mimicking acute infarction. 138th European society of neuroradiology Diagnostic and Interventional ANNUAL MEETING. Neuroradiology. 2015:(57):1-169.
171. Gulsen I, Senol N, Görgülü A. Primary Cranial Epidural Hydatid Cyst: A Rare Location. Journal of Neurological Sciences. 2013;30(4):814-818.
172. Gun E, Etit D, Buyuktalanci DO, Cakalagaoglu F. Unusual locations of hydatid disease: A 10-year experience from a tertiary reference center in Western Turkey. Ann Diagn Pathol. 2017;29:37-40.
173. Guney O, Ozturk K, Kocaogullar Y, Eser O, Acar O. Submandibular and intracranial hydatid cyst in an adolescent. Laryngoscope. 2002;112(10):1857-60.
174. Guo HR, Lu YJ, Bao YH, Zhang TR. Parasellar epidural hydatid cysts. Neurosurgery. 1993;32(4):662-5.
175. Gupta A, Singh S, Madan D. Multiple Intracranial Cystic Brain Lesions: A Diagnostic Dilemma. Pediatr Neurol. 2019;93:56-58.
176. Gupta D, Sharma MR, Shilpakar, SK. Giant hydatid cyst of brain. Nepal Journal of Neuroscience. 2007;4:77.
177. Gupta M, Dhole, TN, Kishore S. A case of disseminated hydatidosis in a 10 year old child. Abstract Number: 268. International Science Symposium on HIV and Infectious Diseases (ISSHID 2019): Infectious diseases: Chennai, India. 12-14 October 2019. BMC Infect Dis. 2020;20;20(1):324.
178. Gupta R, Gupta S, Mahajan B. Multiple Intracranial Hydatid Cysts: A Rare Presentation. JK Science. 2013:15(4):202-204.
179. Gupta R, Sharma SB, Prabhakar G, Mathur P. Hydatid disease in children: Our experience. Formosan Journal of Surgery. 2014:47(6):211-220.
180. Gupta S, Desai K, Goel A. Intracranial hydatid cyst: a report of five cases and review of literature. Neurol India. 1999;47(3):214-7.
181. Gupta SK, Tandon SC, Khanna S, Asthana S. Case report: multiple intracranial hydatid cysts with post-operative dissemination. Clin Radiol. 1991;44(3):203-4.
182. Guvenc G, Özdemir N, Yildrim L. Successful treatment of hydatid cyst into the lateral ventricle with Dowling's technique in an adult patient. Journal of Neurological Sciences (Turkish). 2011;28(2):265-9.
183. Guzel A, Tatli M, Maciaczyk J, Altinors N. Primary cerebral intraventricular hydatid cyst: a case report and review of the literature. J Child Neurol. 2008;23(5):585-8.
184. Hagemann G, Gottstein B, Witte OW. Isolated Echinococcus granulosus hydatid cyst in the CNS with severe reaction to treatment. Neurology. 1999;23;52(5):1100-1.
185. Hajhouji F, Aniba K, Laghmari M, Lmejjati M, Ghannane H, Benali SA. Epilepsy: unusual presentation of cerebral hydatid disease in Children. Pan Afr Med J. 2016;3;25:58.
186. Hamza R, Touibi S, Jamoussi M, Bardi-Bellagha I, Chtioui R. Intracranial and orbital hydatid cysts. Neuroradiology. 1982;22(4):211-4.
187. Hasan ZN, Sagban WJ, Hatim AK, Assad MA. Intracranial hydatid cyst. Clinical features and outcomes of surgical treatment of a series of 8 Iraqi cases. Neurosciences (Riyadh). 2013;18(2):187-9.
188. Ibn Elhadj Z, Boukhris M, Kammoun I, Halima AB, Addad F, Kachboura S. Cardiac hydatid cyst revealed by ventricular tachycardia. J Saudi Heart Assoc. 2014;26(1):47-50.
189. Ihara T, Imai T, Saito H, Tashiro K, Onmura Y. Cerebral alveolar hydatid cyst--case report. Neurol Med Chir (Tokyo). 1991;31(6):342-5.

190. Ijaz L, Mirza B, Nadeem MM, Saleem M. Simultaneous giant hydatid cysts of brain and liver. J Coll Physicians Surg Pak. 2015;25(1):S53-5.
191. Imperato A, Consales A, Ravegnani M, Castagnola E, Bandettini R, Rossi A. Primary Hydatid Cyst of the Brain in a Child: A Case Report. Pol J Radiol. 2016;1;81:578-582.
192. Iplikçioğlu AC, Ozer AF, Benli K, Işik N, Erbengi A. Multiple cerebral hydatid cysts: report of two cases. Br J Neurosurg. 1989;3(2):217-9.
193. Iraci G, Tomazzoli L, Fiore DL, Gerosa MA, Peserico L. Recurrence, with intracranial extension, of an intraorbital hydatid cyst: A clinical follow-up case report. Orbit. 1984;3(1):59-65.
194. Is M, Gezen F, Akyuz F, Aytekin H, Dosoglu M. A 13-year-old girl with a cystic cerebellar lesion: consider the hydatid cyst. J Clin Neurosci. 2009;16(5):712-3.
195. Itumur K, Tamam Y, Karabulut A, Guzel A, Kilic N. Co-occurrence of Cardiac and Cerebral Hydatid Cysts: A Case Report. Scottish Medical Journal 2006 51(3):1-5.
196. Ivanov G. A study of pulmonary hydatid disease in children. 1. Epidemiological and clinical characteristics. Ann Trop Med Parasitol. 1996;90(2):167-71.
197. Ivanov, V. PS273 Cardiac Echinococcosis, Clinical Presentation, Diagnosis and Surgical Treatment. Global Heart. 2016;11(2):e60.
198. Iyigun O, Uysal S, Sancak R, Hokelek M, Uyar Y, Bernay F, Ariturk E. Multiple organ involvement hydatid cysts in a 2-year-old boy. J Trop Pediatr. 2004;50(6):374-6.
199. Izci Y, Tüzün Y, Seçer HI, Gönül E. Cerebral hydatid cysts: technique and pitfalls of surgical management. Neurosurg Focus. 2008;24(6):E15.
200. Jemel N, Gader G, Bahri K, Rkhami M, Badri M, Zammel I. Cerebellar hydatid cyst - a rare case report Cysticka hydatidoza mozecku - zacna kazuistika. Cesk Slov Neurol. 2020;83/116(2):203-4.
201. Joshi MP, Potode RJ, Bhole AM, Joharapurkar SR. Unilobar multiple cerebral hydatid cyst: a rare disease. Neurol India. 2008;56(4):483-4.
202. Kabatas S, Yilmaz C, Cansever T, Gulsen S, Sonmez E, Altinors MN. The management of a complicated brain hydatid cyst: case report. Neurol Neurochir Pol. 2009;43(6):575-8.
203. Kadioğlu HH, Tüzün Y, Kayaoğlu CR, Aydin IH. Primary multiple hydatid cysts localizing in the petroclival area. J Clin Neurosci. 1998;5(4):447-50.
204. Kalaitzoglou I, Drevelengas A, Petridis A, Palladas P. Albendazole treatment of cerebral hydatid disease: evaluation of results with CT and MRI. Neuroradiology. 1998;40(1):36-9.
205. Kamali NI, Huda MF, Srivastava VK. Intraventricular hydatid cyst causing entrapped temporal horn syndrome: Case report and review of literature. Trop Parasitol. 2011;1(2):113-5.
206. Kamath SM, Mysorekar VV, Rao SG, Varma RG. Intraventricular hydatid cyst in a child. Indian J Pathol Microbiol. 2009;52(4):571-2.
207. Kandemirli SG, Cingoz M, Olmaz B, Akdogan E, Cengiz M. Cerebral Hydatid Cyst with Intraventricular Extension: A Case Report. J Trop Pediatr. 2019;1;65(5):514-519.
208. Kanj AH, Fares YH, Yehya RR, Hamzeh FF. Unusual appearance of a cerebral hydatid cyst as a hemorrhagic infarct. Neurosciences (Riyadh). 2010;15(4):275-6.
209. Kanpolat Y, Mertol T, Sekerci Z, Kökeş F. Hydatid cyst of the cavernous sinus. Clin Neurol Neurosurg. 1988;90(2):175-6.
210. Kara ME, Uzun E. Unusual localisations of hydatid cyst and its clinicopathological features. Abstracts. Virchows Archiv. Virchows Arch. 2019;475(1):s301-s302.
211. Karadağ O, Gürelik M, Ozüm U, Göksel HM. Primary multiple cerebral hydatid cysts with unusual features. Acta Neurochir (Wien). 2004;146(1):73-7.
212. Karak PK, Mittal M, Bhatia S, Mukhopadhyay S, Berry M. Isolated cerebral hydatid cyst with pathognomonic CT sign. Neuroradiology. 1992;34(1):9-10.
213. Karakoç ZC, Kasimcan MO, Pipia AP, Tore G, Alberti A, Varcasia A, Sav A, Oruçkaptan H. A life-threatening brainstem compression by cerebral Echinococcus granulosus. Infez Med. 2016;24(1):62-6.
214. Karthigeyan M, Salunke P, Malik P. Cerebellar hydatid. Postgrad Med J. 2019;95(1129):621.
215. Kartikueyan R, Patel SM, Chattopadhyay A, Krishnan P. Primary cerebral hydatid cyst: An unusual cause of very slowly progressive hemiparesis in a child. J Neurosci Rural Pract. 2016;7(4):603-604.
216. Kashikar SV, Lakhkar BN. A boulder in the brain. J Clin Diagn Res. 2013;7(10):2414-5.

217. Kaya U, Ozden B, Türker K, Tarcan B. Intracranial hydatid cysts. Study of 17 cases. J Neurosurg. 1975;42(5):580-4.
218. Kayaoglu CR. Giant hydatid cyst in the posterior fossa of a child: a case report. J Int Med Res. 2008;36(1):198-202.
219. Kemaloğlu S, Ozkan U, Bükte Y, Acar M, Ceviz A. Growth rate of cerebral hydatid cyst, with a review of the literature. Childs Nerv Syst. 2001;17(12):743-5.
220. Keser SH, Selek A, Ece D, Barişik CC, Şensu S, Geçmen GG, Erhan SŞ, Kökten ŞÇ, Barişik NÖ, Gül AE. Review of Hydatid Cyst with Focus on Cases with Unusual Locations. Turk Patoloji Derg. 2017;33(1):30-36.
221. Kessler A, Lipschitz R. Hydatid disease of the brain with unusual radiographic appearances following the use of both negative and positive contrast media. J Fac Radiol. 1958;9(2):106-7.
222. Ketaren RJ, Simca J, Hartoyo V. Silent intracerebral hydatid cyst presenting with atypical neurologic deficits. Journal of the Neurological Sciences 405S. 2019:116542.
223. Khalatbari MR, Brunetti E, Shobeiri E, Moharamzad Y. Calcified Mass on Brain CT in a Teenager with Refractory Seizures. Neuroradiol J. 2014;27(6):691-6.
224. Khaldi M, Mohamed S, Kallel J, Khouja N. Brain hydatidosis: report on 117 cases. Childs Nerv Syst. 2000;16(10-11):765-9.
225. Khan MB, Riaz M, Bari ME. Multiple cerebral hydatid cysts in 8-year-old boy: A case report and literature review of a rare presentation. Surg Neurol Int. 2015;29;6:125.
226. Khattala K, Elmadi A, Rami M, Mahmoudi A, Bouabdallah Y. Enorme kyste hydatique cérébral révélé par un coma [Huge cerebral hydatid cyst revealed by a coma]. Pan Afr Med J. 2012;23;13:64.
227. Khetan P. Unusual multiple intracranial hydatid cyst. 39th Annual Meeting of the International Society for Pediatric Neurosurgery, Goa, India. 2011;27(10):1751-1850.
228. Kia EB, Rahimi H, Sharbatkhori M, Talebi A, Fasihi Harandi M, Mirhendi H. Genotype identification of human cystic echinococcosis in Isfahan, central Iran. Parasitol Res. 2010;107(3):757-60.
229. King TT, Couch RS. The diagnosis of cerebral hydatid disease. Clin Radiol. 1961;12:190-3.
230. Kitis O, Calli C, Yunten N. Report of diffusion-weighted MRI in two cases with different cerebral hydatid disease. Acta Radiol. 2004;45(1):85-7.
231. Kızılca O, Altaş M, Senol U, Oztek MA. Hydatid disease located in the cerebellomedullary cistern. Case Rep Med. 2014;2014:271365.
232. Kocaman S, Ersahin Y, Mutluer S. Cerebral hydatid cysts in children. J Neurosci Nurs. 1999;31(5):270-7.
233. Kojundzic SL, Dolic K, Buca A, Jankovic S, Besenski N. Hydatid Disease with Multiple Organ Involvement: A Case Report. Maced J Med Sci. 2010;3(2):154-1.
234. Köktekir E, Erdem Y, Gökçek C, Karatay M, Yılmaz A, Bayar MA, Sümer S. Calcified intracranial hydatid cyst: case report. Turkiye Parazitol Derg. 2011;35(4):220-3.
235. Kovacs Z, Satan E, Szasz O, Para S. Echinococcosis - Hydatidosis treated in county mures. Acta Microbiol Imm H. 2011;58(1):121-2.
236. Krajewski R, Stelmasiak Z. Cerebral hydatid cysts in children. Child's Nerv Syst. 1991;7:154-155.
237. Kulaçoğlu İH, Oruç MT, Kocaerkek Z, Seçkin S, Coşkun F. Unusual locations of hydatid disease: an evaluation of 77 cases. Turk J Gastroenterol. 2001;12:299-302.
238. Kurtsoy A, Oktem IS, Koç RK, Akdemir H, Menkü A, Tucer B. Successful surgical treatment of a thalamic hydatid cyst with contralateral transcallosal approach. Case report and review of the literature. Pediatr Neurosurg. 1999;31(2):96-9.
239. Lakhdar F, Benzagmout M, Chakour K, Chaoui M el faiz. Multiple and infected cerebral hydatid cysts mimicking brain tumor: Unusual presentation of hydatid cyst. Interdisciplinary Neurosurgery. 2020;22:100802.
240. Lapage CP. Specimen showing part of a hydatid cyst of the brain of a boy, aged 10 Years. Proc R Soc Med. 1914;7:145-6.
241. Li Y, Wang K, Chen L, Zhou J. A giant primary intracerebral ossific lesion caused by the cerebral hydatid disease. Asian J Surg. 2022;45(1):556-558.
242. Limaiem F, Bellil S, Bellil K, Chelly I, Mekni A, Kallel J, Haouet S, Zitouna M, Kchir N. Hydatid cyst of the cranial vault. J Infect Dev Ctries. 2009;15;3(10):807-10.
243. Limaiem F, Bellil S, Bellil K, Chelly I, Mekni A, Khaldi M, Haouet S, Zitouna M, Kchir N. Primary hydatidosis of the central nervous system: a retrospective study of 39 Tunisian cases. Clin Neurol Neurosurg. 2010;112(1):23-8.
244. Lipschitz R. Asymmetry of the skull due to hydatid disease. Br J Radiol. 1960;33:460-1.

245. Llanes EG, Stibal A, Mühlethaler K, Vajtai I, Häusler R, Caversaccio M. Echinococcosis presenting as an otogenic brain abscess: an unusual lesion of the middle ear cleft and temporal lobe. Auris Nasus Larynx. 2008;35(1):115-20.
246. Lobo Z, Psevdos G. Headache in an Uzbekistani Immigrant: A Startling Diagnosis. Infectious Diseases in Clinical Practice. 2019;27(1):62-3.
247. Lotfinia I, Vahedi P, Hadidchi S, Djavadzadegan H. Multiple cerebral hydatid cysts secondary to embolization from intracardiac hydatidosis. Neurosurgery Quarterly. 2007:17(2):134-137.
248. Lunardi P, Missori P, Di Lorenzo N, Fortuna A. Cerebral hydatidosis in childhood: a retrospective survey with emphasis on long-term follow-up. Neurosurgery. 1991;29(4):515-7.
249. Luo K, Luo DH, Zhang TR, Wen H. Primary intracranial and spinal hydatidosis: a retrospective study of 21 cases. Pathog Glob Health. 2013;107(2):47-51.
250. Ma L, Chen DC, Zou SY, Liu YY, Zhou LY, Xiu ZG. Epidemiological characteristics of hepatic echinococcosis, concurrent cerebral echinococcosis, and pulmonary echinococcosis in Ganzi County, Sichuan Province, China. Medicine (Baltimore). 2020;99(15).
251. Maamri K, Cherif I, Trifa A, Nessib N, Elkahla G, Darmoul M. Hydatid cyst in the third ventricle of the brain: case report of an exceptionally rare condition. Childs Nerv Syst. 2022;38(8):1637-1641.
252. Madeo J, Zheng X, Ahmed S, Ramos De Oleo R. Primary cerebral echinococcosis presenting as long-standing generalized weakness. Germs. 2013;1;3(2):63-6.
253. Magoha Ma, Mohan Nk, Musau Ck And Okemwa Mp. Giant solitary cerebral hydatid cyst in a child, complicated with post-operative subdural haematoma: case report and review of literature. East African Medical Journal. 2016:93(111):626-631.
254. Mahmoudi S, Elikaee S, Keshavarz H, Pourakbari B, Mamishi S. Pediatric hydatidosis in Iranian referral pediatrics center. Iran J Parasitol. 2012;7(2):87-91.
255. Majumdar K, Saran RK, Sakhuja P, Jagetia A, Sinha S. Intact protoscolices and hooklets in cytospin preparation of intra-operative cyst fluid allow rapid confirmation of rare cerebral intraventricular hydatid infestation. Cytopathology. 2013;24(4):277-9.
256. Malouf J, Saksouk FA, Alam S, Rizk GK, Dagher I. Hydatid cyst of the heart: diagnosis by two-dimensional echocardiography and computed tomography. Am Heart J. 1985;109(3 Pt 1):605-7.
257. Mancuso P, Noubari BA, Gurusinghe NT. Multiple primary cerebral hydatid cysts: case report. Br J Neurosurg. 1997;11(3):248-9.
258. Mascalchi M, Ragazzoni A, Dal Pozzo G. Pontine hydatid cyst in association with an acoustic neurinoma: MR appearance in an unusual case. AJNR Am J Neuroradiol. 1991;12(1):78-9.
259. McCorkell SJ, Lewall DB. Computed tomography of intracerebral echinococcal cysts in children. J Comput Assist Tomogr. 1985;9(3):514-8.
260. Meera M, Vrushali T, Tanaya, Krunal L. Primary multiple intraventricular hydatid cysts in a child. Trop Parasitol. 2018;8(1):47-49.
261. Menekse G, Ozsoy KM, Daglioglu E, Guzel E, Guzel A, Belen D. Pediatric Giant-Sized Intracerebral Hydatid Cyst: Reports Of Two Cases. Journal of Neurological Sciences. 2012;29(4):841-846.
262. Menkü A, Kulaksizoglu O, Tucer B, Kurtsoy A, Akdemir H. Successful surgical excision of a gigantic cerebral hydatid cyst. Minim Invasive Neurosurg. 2004;47(1):61-4.
263. Menkü A, Kurtsoy A, Tücer B, Durak AC, Akdemir H. Calcified cerebral hydatid cyst following head trauma: case report. Turkish Neurosurgery. 2014;14(1-2):36-40.
264. Miabi Z, Hashemi H, Ghaffarpour M, Ghelichnia R H. Clinicoradiological findings and treatment outcome in patients with intracranial hydatid cyst. Acta Med Iran. 2005;43(5):359-364.
265. Micheli F, Lehkuniec E, Giannaula R, Caputi E, Paradiso G. Calcified cerebral hydatid cyst. Eur Neurol. 1987;27(1):1-4.
266. Miller D, Fleming J. Intracranial hydatid disease, with report of a case. Aust N Z J Surg. 1948;17(4):291-6.
267. Mingde Q, Zhesheng H. Echinococcosis of the central nervous system. Eur Neurol. 1981;20(2):125-31.
268. Mohindra S, Savardekar A, Gupta R, Tripathi M, Rane S. Varied types of intracranial hydatid cysts: radiological features and management techniques. Acta Neurochir (Wien). 2012;154(1):165-72.
269. Molavipour A, Javan H, Moghaddam AA, Dastani M, Abbasi M, Ghahramani S. Combined medical and surgical treatment of intracardiac hydatid cysts in 11 patients. J Card Surg. 2010;25(2):143-6.
270. Money RA. Hydatid disease of the brain; with a report of two cases. Br J Surg. 1958;18;45(193):454-7.

271. Moskopp D, Lotterer E. Concentrations of albendazole in serum, cerebrospinal fluid and hydatidous brain cyst. Neurosurg Rev. 1993;16(1):35-7.
272. Müfit K, Nejat I, Mercan S, Ibrahim K, Mete UY, Yüksel K. Growth of multiple hydatid cysts evaluated by computed tomography. J Clin Neurosci. 1998;5(2):215-7.
273. Mufti T. Intracranial hydatidosis. J Pak Med Assoc. 1983;33(5):115-8.
274. Mushtaque M, Mir MF, Malik AA, Arif SH, Khanday SA, Dar RA. Atypical localizations of hydatid disease: experience from a single institute. Niger J Surg. 2012;18(1):2-7.
275. Mussa T, Bijan Z, Mohammad Hadi B, Zahra Z. Hydatid Cyst of the Foramen Magnum. Neurosurgery Quarterly. 2005;15(2):110-112.
276. Muthusubramanian V, Pande A, Vasudevan MC, Ravi R. Surgical management of brainstem hydatid cyst--an unusual site. Surg Neurol. 2009;71(1):103-6.
277. Naderzadeh A, Ghanim SM, Keikhosravi E, Shojaeian R. Childhood refractory headache: Alarming sign of hydatid disease in endemic area. Journal of Pediatric Surgery Case Reports. 2020;61:1-4.
278. Naim-ur-Rehman. Hydatid disease of the central nervous system. J Pak Med Assoc. 1980;30(8):186-9.
279. Nashibi M, Tafrishinejad A, Hussain Khan Z. Deep seated cerebral hydatid cyst and its anesthetic considerations: A Case Report. Arch Neurosci. 2020;7(1):e100044.
280. Ndondo AP, Fieggen G, Wilmshurst JM. Hydatid disease of the spine in South African children. J Child Neurol. 2003;18(5):343-6.
281. Neeraj A, Karam C, Avninder SP. Gigantic Intracranial Hydatid Cysts: An Unusual Case Report. Journal of Pediatric Neurology. 2006;4(2):115-119.
282. Negovetic L, Lupret V, Smiljanic D, Arsenic B. Cranial vault and gigantic intracranial hydatid cyst in a young woman. Neurosurgery. 1990;27(3):480-1.
283. Nejad MR, Hoseinkhan N, Nazemalhosseini E, Cheraghipour K, Abdinia E, Zali MR. An analysis of hydatid cyst surgeries in patients referred to hospitals in Khorram-Abad, Lorestan during 2002-06. Iranian Journal of Parasitology. 2007;(2):29-33.
284. Nemati A, Kamgarpour A, Rashid M and Nazari SS. Giant Cerebral Hydatid Cyst in a Child- A Case Report and Review of Literature. BJMP. 2010;3(3):a338.
285. Nie D, Xia L, Chen J, Shi W, Sun G, Guo J. Teaching NeuroImages: Giant cystic echinococcosis with unusual imaging manifestations. Neurology. 2017;6;88(23):e234-e235.
286. Nourmand A. Hydatid cysts in children and youths. Am J Trop Med Hyg. 1976;25(6):845-7.
287. Ntusi NA, Horsfall C. Severe disseminated hydatid disease successfully treated medically with prolonged administration of albendazole. QJM. 2008;101(9):745-6.
288. Nurchi G, Floris F, Montaldo C, Mastio F, Peltz T, Coraddu M. Multiple cerebral hydatid disease: case report with magnetic resonance imaging study. Neurosurgery. 1992;30(3):436-8.
289. O'Malley A, Sekaran L, Phiri D and Dattani M. An unusual infective cause for strokes. European stroke organisation conference: Abstracts. European Stroke Journal. 2018;3(1):3-204.
290. Obrador Alcalde S. Some neurosurgical features of the tuberculomas and parasitic cysts of the brain. Folia Psychiatr Neurol Neurochir Neerl. 1951;54(4-5):295-9.
291. Obrador S, Urquiza P. Two cases of cerebral abscess of unusual nature; tuberculous abscess and suppurated hydatid cyst. J Neurosurg. 1948;5(6):572-6.
292. Ökten AI, Ergün R & Gezercan Y. Primary intracranial extradural hydatid cyst localized in the supra-and infra-tentorium. Acta Parasit. 2006;51:309-310.
293. Onal C, Barlas O, Orakdögen M, Hepgül K, Izgi N, Unal F. Three unusual cases of intracranial hydatid cyst in the pediatric age group. Pediatr Neurosurg. 1997;26(4):208-13.
294. Onal C, Erguvan-Onal R, Yakinci C, Karayol A, Atambay M, Daldal N. Can the requirement of a diversion procedure be predicted after an uncomplicated intracranial hydatid cyst surgery? Pediatr Neurosurg. 2006;42(6):383-6.
295. Onal C, Unal F, Barlas O, Izgi N, Hepgul K, Turantan MI, Canbolat A, Turker K, Bayindir C, Gokay HK, Kaya U. Long-term follow-up and results of thirty pediatric intracranial hydatid cysts: half a century of experience in the Department of Neurosurgery of the School of Medicine at the University of Istanbul (1952-2001). Pediatr Neurosurg. 2001;35(2):72-81.
296. Onal C, Yakinci C, Erten F, Erguvan R, Cayli S, Gül A, Aydin E. Supratentorial hydatid cyst with cerebellar signs: a rare case of diaschisis. Childs Nerv Syst. 2001;17(12):746-9.
297. Orman DN, LE Roux PA. Cerebral hydatid disease: a radiological review. S Afr Med J. 1968;12;42(39):1048-51.

298. Özdemir Hl, Karbuz A, Kocabaş BA, Yahşi A, Erat T, Bingöl-Koloğlu M, Fitöz S, Tutar E, Çiftçi E, Ince E. P270 Clinical characteristics of childhood hydatid disease: a single tertiary centre experience from turkey. Archives of Disease in Childhood. 2017;102:A138.
299. Ozgen T, Erbengi A, Bertan V, Sağlam S, Gürçay O, Pirnar T. The use of computerized tomography in the diagnosis of cerebral hydatid cysts. J Neurosurg. 1979;50(3):339-42.
300. Palani A. Partially calcified giant intracerebral hydatid cyst in a pediatric child. Neurol India. 2012;60(2):260-2.
301. Panda NB, Batra Y, Mishra A, Dhandapani S. A giant intracranial hydatid cyst in a child: Intraoperative anaesthetic concerns. Indian J Anaesth. 2014;58(4):477-9.
302. Pandey S, Pandey D, Shende N, Sahu A, Sharma V. Cerebral intraventricular echinococcosis in an adult. Surg Neurol Int. 2015;18;6:138.
303. Patrikar DM, Mitra KR, Bhutada VR. Cerebral hydatid disease. Australas Radiol. 1993;37(2):226-7.
304. Pau A, Perria C, Turtas S, Brambilla M, Viale G. Long-term follow-up of the surgical treatment of intracranial coenurosis. British Journal of Neurosurgery. 1990;4(1):39-43.
305. Per H, Gumus H, Kumandas S, Tucer BL, Yikilmaz A, Menku A, Coskun A, Kurtsoy A. Gigantic cerebral hydatid cysts in childhood. Ann Saudi Med. 2007;27(1):60-1.
306. Per H, Kumandaş S, Gümüş H, Kurtsoy A. Primary soliter and multiple intracranial cyst hydatid disease: report of five cases. Brain Dev. 2009;31(3):228-33.
307. Perde F, Enachescu CI & Ceaușu M Incidental finding of heart and brain echinococcosis in a patient with carbon monoxide poisoning. Romanian Journal of Legal Medicine. 2016;24:190-193.
308. Peter JC, Domingo Z, Sinclair-Smith C, de Villiers JC. Hydatid infestation of the brain: difficulties with computed tomography diagnosis and surgical treatment. Pediatr Neurosurg. 1994;20(1):78-83.
309. Phillips G. Primary cerebral hydatid cysts. J Neurol Neurosurg Psychiatry. 1948;11(1):44-52.
310. Pillai AK, Pillai MV, Vohra S & Oommen EB. Multiple central nervous system hydatidosis secondary to cardiac echinococcosis. Indian Journal of Radiology and Imaging. 2004;14(1):81-83.
311. Polat G, Ogul H, Sengul G. Hydatidosis Following Giant Cerebral Hydatid Cyst Operation. World Neurosurg. 2018;118:14-15.
312. Popli MB, Khudale B. Primary multiple hydatid cysts of the brain. Australas Radiol. 1998;42(1):90-1.
313. Prati G, Gatti G, Belgrano M, Pinamonti B, Rauber E, Gripshi F, Pappalardo A, Sinagra G. Disseminated echinococcosis: follow your heart. J Cardiovasc Med (Hagerstown). 2016;17(2):e146-e148.
314. Pulavarty P, Korde P, Rathod S, Patnaik J, Domakunti R, Singh SP. Primary solitary hydatid disease of brain in a 16-year-old girl: a case report. Pan Afr Med J. 2022 Jul 12;42:195.
315. Qadri SK, Hamdani NH, Bhat AR, Lone MI. Unusual presentation of an intraventricular hydatid cyst as a bleeding cystic tumor: A case report and brief review. Asian J Neurosurg. 2017;12(2):324-327.
316. Qiang Z, Xiumin H, Mingfei Y. Cerebral cystic echinococcosis among children in Qinghai, china: a case series. Southeast Asian Journal of Tropical Medicine and Public Health. 2019;50(4):621-627.
317. Radmenesh F, Nejat F. Primary Cerebral Hydatid Cyst: Two Cases Report. Iran J Pediatr. 2008;18(1):83-86.
318. Ramosaço E, Kolovani E, Ranxha E, Vyshka G. Primary multiple cerebral hydatid cysts in an immunocompetent, low-risk patient. ID Cases. 2020;20;21:e00882.
319. Randev S, Gupta VK, Kumar P, Mahajan V, Angurana SK, Guglani V. Brain Hydatid in a Child. J Pediatr. 2018;199:280.
320. Ranjbar-Bahadori Sh, Lotfollahzadeh S, Vaezi G and Eslami A. Epidemiological Study of the Human Cystic Echinococcosis in Iran. Research Journal of Parasitology 2008;3(4):130-136.
321. Raul S, Bajpayee CP. Primary hydatid cyst in cerebellopontine angle. Med J. Armed Forces India. 1991;47(3):232-4.
322. Ravanbakhsh N, Rabiee N, Ahmadi J. Primary Solitary Hydatid Cyst of Brain in a 12-Year-Old Boy: A Case Report. Iran J Parasitol. 2019;14(4):668-673.
323. Ray M, Singhi PD, Pathak A, Khandelwal NK. Primary multiple intracerebral echinococcosis in a young child. J Trop Pediatr. 2005;51(1):59-61.
324. Raynham OW, Mulwafu W, Fagan JJ. Hydatid disease of the skull base: report of three cases and a literature review. Skull Base. 2009;19(2):171-5.
325. Razzaq AA, Hashim AS. Multiple cerebral hydatid cysts: a surgical challenge. J Pak Med Assoc. 2000;50(1):35-7.
326. Rebhandl W, Turnbull J, Felberbauer FX, Tasci E, Puig S, Auer H, Paya K, Kluth D, Tasci O, Horcher E. Pulmonary echinococcosis (hydatidosis) in children: results of surgical treatment. Pediatr Pulmonol. 1999;27(5):336-40.

327. Reddy CC, Reddy MM, Sarada P, Kumari GS, Hari N, Sarma HN, Yellappa K. Hydatid disease in children. Indian J Pediatr. 1979;46(382):397-400.
328. Reddy DR, Murthy JM. Parasitic intracranial space-occupying lesions in children in India. Childs Nerv Syst. 1986;2(5):244-7.
329. Regaieg K, Bahloul M, Turki O, Kammoun B, Toumi N, Bouaziz M. Giant Intracranial Cystic Lesion in a Child. Wilderness Environ Med. 2018;29(4):546-548.
330. Rhodes PL. Unusual case of hydatid cyst of the brain. Br Med J. 1954;25;2(4890):739.
331. Romodanov AP. Variations in the clinical course of various types of tumours located in the cerebral hemispheres in children. Symposium: Problemy Neirokhirurgii. 1959;4:59-66.
332. Rudwan MA, Khaffaji S. CT of cerebral hydatid disease. Neuroradiology. 1988;30(6):496-9.
333. Rumboldt Z, Jednačak H, Talan-Hranilović J, Rumboldt T, Kalousek M. Unusual appearance of a cisternal hydatid cyst. American journal of neuroradiology. 2003;24(1):112-4.
334. Sabouni F, Ferdosian F, Mamishi S, Nejat F, Monnajemzadeh M, Rezaei N. Multiple organ involvement with hydatid cysts. Iran J Parasitol. 2010;5(2):65-70.
335. Sadashiva N, Shukla D, Devi BI. Rupture of Intraventricular Hydatid Cyst: Camalote Sign. World Neurosurg. 2018;110:115-116.
336. Sadjjadi SM, Mikaeili F, Karamian M, Maraghi S, Sadjjadi FS, Shariat-Torbaghan S, Kia EB. Evidence that the Echinococcus granulosus G6 genotype has an affinity for the brain in humans. Int J Parasitol. 2013;43(11):875-7.
337. Safavi M, Dabiri S. Calcified cerebral hydatid cyst presenting with seizure. Arch Iran Med. 2019;1;22(2):102-103.
338. Şahin-Akyar G. Computed tomography and magnetic resonance imaging findings in cerebral hydatid disease. Radiography. 2002;8(4):251-258.
339. Salunke P, Patra DP, Mukherjee KK. Delayed cerebral vasospasm and systemic inflammatory response syndrome following intraoperative rupture of cerebral hydatid cyst. Acta Neurochir (Wien). 2014;156(3):613-4.
340. Samadian M, Mousavinejad SA, Jabbari A, Tavassol HH, Karimi P, Almagro K, Rezaei O, Borghei-Razavi H. Third ventricle hydatid cyst: A rare case report and review of the literature. Clin Neurol Neurosurg. 2020;198:106218.
341. Samiy E, Zadeh FA. Cranial and intracranial hydatidosis, with special reference to roentgen-ray diagnosis. J Neurosurg. 1965;22(5):425-33.
342. Sandhu P, Saggar K, Sodhi KS. Neurological picture. Multiple hydatid cysts of the brain after surgery. J Neurol Neurosurg Psychiatry. 2000;68(1):97.
343. Sanlı AM, Türkoğlu E, Kertmen H, Gürer B. Hydatid cyst of the ambient cistern radiologically mimicking an arachnoid cyst. J Neurosurg Pediatr. 2012;10(3):186-8.
344. Saqui AE, Aggouri M, Benzagmout M, Chakour K, Faizchaoui ME. Kyste hydatique de la fosse cérébrale postérieure [Hydatid cyst of the posterior fossa]. Pan Afr Med J. 2017;9;26:133.
345. Sarmast AH, Showkat HI, Shah NF, Mujtaba B, Malik AA, Malik NK, Parray FQ. Hydatids everywhere: A 15-year experience of unusual locations of the disease in an endemic area. J Res Med Sci. 2016; 8;21:25.
346. Schijman E. Hydatid cysts of the posterior fossa. Neurosurgery. 1985;17(6):1014-5.
347. Seckin H, Yagmurlu B, Yigitkanli K, Kars HZ. Metabolic changes during successful medical therapy for brain hydatid cyst: case report. Surg Neurol. 2008;70(2):186-9.
348. Sen N, Laha D, Gangopadhyay PK, Mohanty BC. Young girl with multiple intracranial hydatid cyst. Ann Neurosci. 2012;19(2):96-8.
349. Sener E, Kurt A. Hydatid cyst disease in children: 10-years experience at two tertiary centers from Northeast Anatolia of Turkey. Ann Med Res 2020;27(4):1103-8
350. Sener RN. Thalamic hydatid cyst: contrast-enhanced MR imaging findings. Comput Med Imaging Graph. 1996;20(5):395-8.
351. Şengül G, Çakir M, Çalikoğlu Ç, Duman S, Zeynal M, Duman A. Cerebral hydatid disease: clinical analysis of ten cases. Journal of Neurological Sciences 2012;29(4):754-760.
352. Shafiei R, Ghatee MA, Jafarzadeh F, Javanshir Z, Karamian M. Genotyping and phylogenetic analysis of unusually located hydatid cysts isolated from humans in north-east Iran. J Helminthol. 2019;23;94:e64.
353. Shafiei R, Raeghi S, Jafarzadeh F, Najjari M, Ghatee MA, Shokri A. Three cases of brain hydatidosis in North Khorasan, Iran. Clin Case Rep. 2022;22;10(7):e6095.
354. Shahriarirad R, Erfani A, Eskandarisani M, Rastegarian M, Sarkari B. Uncommon Locations of Cystic Echinococcosis: A Report of 46 Cases from Southern Iran. Surg Res Pract. 2020;2020:2061045.

355. Shakeri M, Vahedi P. Cerebral Echinococcosis in the Children and Young Adults. Neurosurgery Quarterly. 2007;17(3):241-244.
356. Sharma A, Abraham J. Multiple giant hydatid cysts of the brain. Case report. J Neurosurg. 1982;57(3):413-5.
357. Sharma AK, Diyora B, Badhe P, Nayak N, Patankar P, Chopra G. Neurological picture. Primary multiple calvarial hydatid cysts--a rare occurrence. J Neurol Neurosurg Psychiatry. 2011;82(3):349-50.
358. Sharma M, Sehgal R, Fomda BA, Malhotra A, Malla N. Molecular characterization of Echinococcus granulosus cysts in north Indian patients: identification of G1, G3, G5 and G6 genotypes. PLoS Negl Trop Dis. 2013;13;7(6):e2262.
359. Sharma SC, Ray RC. Primary hydatid cyst of the brain in an adult: report of a case. Neurosurgery. 1988;23(3):374-6.
360. Sharma V, Sharma A, Sharma M, Sharma A, Khajuria A. Primary intracranial multiple hydatid cysts in an adult. International Journal of Medicine and Public Health. 2015;5(3):247-249.
361. Sherwani RK, Abrari A, Jayrajpuri ZS, Srivastava VK. Intracranial hydatidosis. Report of a case diagnosed on cerebrospinal fluid cytology. Acta Cytol. 2003;47(3):506-8.
362. Sheves A, Fuxman Y, Gazer B, Shmueli M, Van Buren J, Ben-Shimol S, Assi Z. Treatment of cystic echinococcosis in children: A single center experience. Pediatr Infect Dis J. 2023;1;42(3):175-179.
363. Shin DH, Jo HC, Kim JH, Jun KI, Park WB, Kim NJ, Choi MH, Kang CK, Oh MD. An Imported Case of Disseminated Echinococcosis in Korea. Korean J Parasitol. 2019;57(4):429-434.
364. Shirmen O, Batchuluun B, Lkhamjav A, Tseveen T, Munkhjargal T, Sandag T, Lkhagvasuren E, Yanagida T, Nishikawa Y, Ito A. Cerebral cystic echinococcosis in Mongolian children caused by Echinococcus canadensis. Parasitol Int. 2018;67(5):584-586.
365. Shukla-Dave A, Gupta RK, Roy R, Husain N, Paul L, Venkatesh SK, Rashid MR, Chhabra DK, Husain M. Prospective evaluation of in vivo proton MR spectroscopy in differentiation of similar appearing intracranial cystic lesions. Magn Reson Imaging. 2001;19(1):103-10.
366. Shukla S, Trivedi A, Singh K, Sharma V. Craniospinal hydatidosis: Report of three cases. Journal of Pediatric Neurosciences. 2008:3(2):146-149.
367. Siddiqui MA, Rizvi SW, Rizvi SA, Ahmad I, Ullah E. Atypical multifocal hydatid disease of cranial vault: simultaneous orbital and extradural meningeal involvement. Emerg Radiol. 2010;17(5):427-30.
368. Sierra J, Oviedo J, Berthier M, Leiguarda R. Growth rate of secondary hydatid cysts of the brain. Case report. J Neurosurg. 1985;62(5):781-2.
369. Şimşek S. Hydatid cyst in the teres major muscle and brain. Rev Soc Bras Med Trop. 2022;24;55:e03172022.
370. Singounas EG, Leventis AS, Sakas DE, Hadley DM, Lampadarios DA, Karvounis PC. Successful treatment of intracerebral hydatid cysts with albendazole: case report and review of the literature. Neurosurgery. 1992;31(3):571-4.
371. Siyadatpanah A, Brunetti E, Emami Zeydi A, Moghadam YD, Agudelo Higuita NI. Cerebral Cystic Echinococcosis. Case Rep Infect Dis. 2020;29;2020:1754231.
372. Skuhala T, Trkulja V, Runje M, Balen-Topić M, Vukelić D, Desnica B. Combined Albenazole-Praziquantel Treatment in Recurrent Brain Echinococcosis: Case Report. Iran J Parasitol. 2019;14(3):492-496.
373. Slim MS, Akel SR. Hydatidosis in childhood. Prog Pediatr Surg. 1982;15:119-29.
374. Smita P and Nair AGA. Tale of two cysties: intra orbital hydatid cyst with intracranial extension. Meeting of the International Neuro-Ophthalmology Society, Singapore. Neuro-ophthalmology. 2012;36(S1):1-65.
375. Stoicescu R, Mihai CM, Catrinoiu D, Balasa A, Mihai L, Cuzic V, Sirbu R, Negreanu T, Arcus M. R2240 - Therapeutical approach in hydatic cyst disease with multiple location. 19th ECCMID, Abstracts. Clinical Microbiology and Infection. 2009;15(4):S613-S677.
376. Sureka J, Sarawagi R, Eapen A, Keshava SN, Vedantam R. Skull base hydatid cyst with intracranial extension presenting as vocal cord palsy: a case report. Br J Radiol. 2010;83(987):e67-9.
377. Svrckova P, Nabarro L, Chiodini PL, Jäger HR. Disseminated cerebral hydatid disease (multiple intracranial echinococcosis). Pract Neurol. 2019;19(2):156-163.
378. Taghipoor M, Razmkon A. Neurological picture. Huge primary intracranial hydatid cysts. J Neurol Neurosurg Psychiatry. 2009;80(10):1149.
379. Taghipour M, Moin H, Zamanizadeh B, Kamkarpour A, Esmaeeli M, Haghnegahdar A, Zare Z, Malekpour B. Foramen Magnum Surgery: Experience With 22 Cases. Neurosurgery Quarterly. 2006;16(2):96-9.

380. Taghipour M, Saffarrian A, Ghaffarpasand F, Azarpira N. Dumbbell-Shape Hydatid Cyst of Meckel Cave Extending to Cerebellopontine Angle and Middle Fossa; Surgical Technique and Outcome of Rare Case. World Neurosurg. 2017;104:1049.
381. Talan-Hranilovic J, Sajko T, Negovetic L, Lupret V, Kalousek M. Cerebral cysticercosis and echinococcosis: a preoperative diagnostic dilemma. Arch Med Res. 2002;33(6):590-4.
382. Tanki H, Singh H, Raswan US, Bhat AR, Kirmani AR, Ramzan AU. Pediatric Intracranial Hydatid Cyst: A Case Series with Literature Review. Pediatr Neurosurg. 2018;53(5):299-304.
383. Tapia E O, Vidal T A, Antonio P L. Hidatidosis cerebral: aspectos clínicos e imagenológicos. Reporte de 4 casos [Brain hydatidosis: report of four cases]. Rev Med Chil. 2012;140(3):358-63.
384. Taslakian B, Darwish H. Intracranial hydatid cyst: imaging findings of a rare disease. BMJ Case Rep. 2016 Sep 12;2016:bcr2016216570.
385. Tatli M, Guzel A, Altinors N. Large primary cerebral hydatid cysts in children. Neurosciences (Riyadh). 2006;11(4):318-21.
386. Teke M, Göçmez C, Hamidi C, Gündüz E, Göya C, Çetinçakmak MG, Hattapoğlu S, Durmaz MS. Imaging features of cerebral and spinal cystic echinococcosis. Radiol Med. 2015;120(5):458-65.
387. Teymoorian GA, Bagheri F. Hydatid cyst of the skull: report of four cases. Radiology. 1976;118(1):97-100.
388. Thakur LC, Anand KS. Brain echinococcal disease in Pondicherry. J Trop Med Hyg. 1993;96(4):256-8.
389. Thakur SH, Joshi PC, Kelkar AB, Seth N. Unusual presentation of hydatid cyst - ruptured intraventricular hydatid. Indian J Radiol Imaging. 2017;27(3):282-285.
390. Todorov T, Vutova K, Petkov D, Balkanski G. Albendazole treatment of multiple cerebral hydatid cysts: case report. Trans R Soc Trop Med Hyg. 1988;82(1):150-2.
391. Tokpanov S, Dosmagambetov S, Kotlobovskiy V. Laparoscopic treatment of liver hydatid disease in cases of cyst rupture. Surgical Endoscopy and Other Interventional Techniques. 2014;28(1):364.
392. Topal U, Parlak MÜFİT, Kihç E, Sivri Z, Sadikolu MY, Tuncel E. European Radiology. 1995;5:244-7.
393. Tor M, Atasalihi A, Altuntas N, Sulu E, Senol T, Kir A, Baran R. Review of cases with cystic hydatid lung disease in a tertiary referral hospital located in an endemic region: a 10 years' experience. Respiration. 2000;67(5):539-42.
394. Trivedi, A, Shukla, S, Singh, K, Sharma, V. Giant intracranial hydatid cyst. Journal of Pediatric Neurosciences. 2007;2(2):72.
395. Trueba-Argamasilla AA, Iborra-Bendicho MA, Simón-Páez M, Ros-de San Pedro J, Segovia-Hernández M. Cerebral echinococcosis: Case report and literature review. Enferm Infecc Microbiol Clin (Engl Ed). 2023;41(2):107-110.
396. Tsitouridis J, Dimitriadis AS, Kazana E. MR in cisternal hydatid cysts. AJNR Am J Neuroradiol. 1997;18(8):1586-7.
397. Turan Y, Yilmaz T, Göçmez C, Kamaşak K, Kemaloğlu S, Tekin R, Hattapoğlu S, Bozkaya H, Çalişkan A, Ceviz A. Assessment of cases with intracranial hydatid cyst: A 23-year experience. J.Neurol.Sci. 2014;31(1):090-098.
398. Turgut M, Benli K, Eryilmaz M. Secondary multiple intracranial hydatid cysts caused by intracerebral embolism of cardiac echinococcosis: an exceptional case of hydatidosis. Case report. J Neurosurg. 1997;86(4):714-8.
399. Turgut M. Primary intracranial extradural hydatid cyst extending above and below the tentorium. Br J Neurosurg. 1997;11(6):587.
400. Turgut M. The role of mebendazole in the surgical treatment of central nervous system hydatid disease. Br J Neurosurg. 1998;12(3):289.
401. Türkoğlu E, Demirtürk N, Tünay H, Akıcı M, Öz G, Baskin Embleton D. Evaluation of Patients with Cystic Echinococcosis. Turkiye Parazitol Derg. 2017;41(1):28-33.
402. Turkoglu OF, Solaroglu I, Tun K, Beskonakli E, Taskin Y. Secondary infection of intracranial hydatid cyst with Clostridium ramosum. Childs Nerv Syst. 2005;21(11):1004-7.
403. Tuzun Y, Kadioglu HH, Izci Y, Suma S, Keles M, Aydin IH. The clinical, radiological and surgical aspects of cerebral hydatid cysts in children. Pediatr Neurosurg. 2004;40(4):155-60.
404. Tuzun Y, Sengul G, Sili M, Izci Y. Pediatric intracranial hydatid cysts. 22ndCongress of European Society for Pediatric Neurosurgery, Belek – Antalya, Turkey. 2010;26(4):545-592.
405. Tuzun Y, Solmaz I, Sengul G, Izci Y. The complications of cerebral hydatid cyst surgery in children. Childs Nerv Syst. 2010 Jan;26(1):47-51.
406. Tzili N, Ahbeddou S, Ahmimech J, Abboud H, Boutarbouch M, El Hassan A, Berraho A. Swollen eyelid reveals multiple intracranial hydatid cysts associated with a palpebral cyst. J Fr Ophtalmol. 2016;39(2):210-2.
407. Uğur HC, Attar A, Bağdatoğlu C, Erdoğan A, Egemen N. Secondary multiple intracranial hydatid cysts caused by intracerebral embolism of cardiac echinococcosis. Acta Neurochir (Wien). 1998;140(8):833-4.

408. Umerani MS, Abbas A, Sharif S. Intra cranial hydatid cyst: A case report of total cyst extirpation and review of surgical technique. J Neurosci Rural Pract. 2013;4(1):S125-8.
409. Vahedi MA, Vahedi ML. Demographics of patients with surgical and nonsurgical cystic echinococcosis in East Azerbaijan from 2001 to 2012. Pak J Biol Sci. 2012;15(4):186-91.
410. Valkanov St, Chakarov I, Popkharitov Ts, Popkharitov A, Chakarova B, Chakarova P, Valkanov P, Petrov B, Mindov I. Surgical treatment of hydatid disease of central nervous system – our experience and review of the literature. Trakia Journal of Sciences. 2016;4:50-55.
411. Vaquero J, Jiménez C, Martínez R. Growth of hydatid cysts evaluated by CT scanning after presumed cerebral hydatid embolism. Case report. J Neurosurg. 1982;57(6):837-8.
412. Vatansever M, Biliciler B, Aladağ MA, Colak A. A huge cerebral hydatid cyst associated with small liver cyst: a comparison of growth rates and sizes. Neurosurg Rev. 1996;19(2):123-6.
413. Vidhate MR, Singh D, Sharma P, Singh MK. Cerebral hydatid cyst showing pathognomonic daughter cysts. Ann Indian Acad Neurol. 2011;14(3):217-8.
414. Vikas S, Preety S, Sanjeev P. Cerebral hydatid cyst: A case report. Acta Med Int 2016;3:207-9
415. Villarejo F, Blazquez MG, Arcas J, Pascual-Castroviejo I, Esteban F. Hydatid cyst of the posterior fossa: case report. Neurosurgery. 1983;12(2):228-9.
416. Vishal K, Vibhuti. Neuroimage--hydatid cyst of brain. J Assoc Physicians India. 2010;58:173.
417. Wani AA, Ramzan AU, Nizami FA, Malik NK, Dar B, Kumar A. Subdural hydatid cyst presenting as recurrent subdural hygroma. Asian J Neurosurg. 2016;11(3):322.
418. Wani NA, Kosar TL, Khan AQ, Ahmad SS. Multidetector-row computed tomography in cerebral hydatid cyst. J Neurosci Rural Pract. 2010;1(2):112-4.
419. Wani NA, Kousar TL, Gojwari T, Robbani I, Singh M, Ramzan A, Khan Q, Kirmani A, Wani A. Computed tomography findings in cerebral hydatid disease. Turk Neurosurg. 2011;21(3):347-51.
420. Waziri TM, Rao Bollineni V, Kadi R, De Mey J. A Giant Intracranial Hydatid Cyst in an Eleven-Year-Old Boy. J Belg Soc Radiol. 2017;101(1):6.
421. Yadav VK, Sudhakar SV, Panwar J. Pathognomonic MRI and MR spectroscopy findings in cerebral hydatid cyst. Acta Neurol Belg. 2016;116(3):353-5.
422. Yaghoobi MH, Sabahi MM, Zibaei M. Imaging features of the lungs hydatid cyst disseminated into the brain and spleen. Radiol Case Rep. 2019;22;14(8):903-5.
423. Yaka U, Aras Y, Aydoseli A, Akcakaya MO, Sencer A, Imer M, Hepgul K. Primary multiple cerebral hydatid disease: still symptomatic despite pathologically confirmed death of the cyst. Turk Neurosurg. 2013;23(4):505-8.
424. Yang YR, Sun T, Zhang JZ, McManus DP. Molecular confirmation of a case of multiorgan cystic echinococcosis. J Parasitol. 2006;92(1):206-8.
425. Yasha TC, Shibu P, Srikanth SG, Shankar SK. 13-year-old boy with cerebellar cystic mass. Neurol India. 2006;54(1):100-1.
426. Yasim A, Ustunsoy H, Gokaslan G, Hafız E, Arslanoglu Y. Cardiac Echinococcosis: A Single-Centre Study with 25 Patients. Heart Lung Circ. 2017;26(2):157-163.
427. Yavuzer D, Ergen C, Dalbayrak S, Yilmaz T, Karadayi N. Cerebral hydatid cyst: case report. Virchows Arch. 2009;455(1):S1-S482.
428. Yikilmaz A, Durak AC, Mavili E, Donmez H, Kurtsoy A, Kontas O. The role of diffusion-weighted magnetic resonance imaging in intracranial cystic lesions. Neuroradiol J. 2009;20;21(6):781-90.
429. Yilmaz N, Kiymaz N, Etlik O, Yazici T. Primary hydatid cyst of the brain during pregnancy. Neurol Med Chir (Tokyo). 2006;46(8):415-7.
430. Yilmaz Y, Kösem M, Ceylan K, Köseoglu B, Yalçinkaya I, Arslan H, Güneş M, Söylemez O. Our experience in eight cases with urinary hydatid disease: a series of 372 cases held in nine different clinics. Int J Urol. 2006;13(9):1162-5.
431. Yilmazlar S, Aksoy K. Approach via the floor of the fourth ventricle for hydatid cyst of the pons. Pediatr Neurosurg. 1999;31(6):326-9.
432. Yolasigmaz, A, Reiterová K, Turk M, Reyhan E, Bozdag A, Karababa A, Altintas Nu, Altintas Na. Comparison of serological and clinical findings in Turkish patients with cystic echinococcosis. Helminthologia. 2006;43(4):220-225.
433. Yüceer N, Gökalp HZ. Periventricular hydatid cyst. J Neurosurg Sci. 1998;42(3):173-5.

434. Yüceer N, Güven MB, Yilmaz H. Multiple hydatid cysts of the brain: a case report and review of the literature. Neurosurg Rev. 1998;21(2-3):181-4.
435. Yurt A, Avci M, Selçuki M, Ozer F, Camlar M, Uçar K, Taşli F, Altinörs N. Multiple cerebral hydatid cysts. Report of a case with 24 pieces. Clin Neurol Neurosurg. 2007;109(9):821-6.
436. Zahawi HM, Hameed OK, Abalkhail AA. The possible role of the age of the human host in determining the localization of hydatid cysts. Ann Trop Med Parasitol. 1999;93(6):621-7.
437. Zahed HM, Mizanur RM, Mohammad BS. Cranial hydatid abscess. Trop Doct. 2010;40(4):255-6.
